# Efficacy and Safety of HER2-Targeted Agents for Breast Cancer with HER2-Overexpression: A Network Meta-Analysis

**DOI:** 10.1371/journal.pone.0127404

**Published:** 2015-05-20

**Authors:** Qiuyan Yu, Zhenli Zhu, Yan Liu, Jun Zhang, Ke Li

**Affiliations:** Department of Public Health, Shantou University Medical College, No.22 Xinling Road, Shantou, Guangdong 515041, China; Shanghai Jiao Tong University School of Medicine, CHINA

## Abstract

**Background:**

Clinical trials of human epidermal growth factor receptor 2 (HER2)-targeted agents added to standard treatment have been efficacious for HER2-positive (HER2+) advanced breast cancer. To our knowledge, no meta-analysis has evaluated HER2-targeted therapy including trastuzumab emtansine (T-DM1) and pertuzumab for HER2-positive breast caner and ranked the targeted treatments. We performed a network meta-analysis of both direct and indirect comparisons to evaluate the effect of adding HER2-targeted agents to standard treatment and examined side effects.

**Methods:**

We performed a Bayesian-framework network meta-analysis of randomized controlled trials to compare 6 HER2-targeted treatment regimens and 1 naïve standard treatment (NST, without any-targeted drugs) in targeted treatment of HER2+ breast cancer in adults. These treatment regimens were T-DM1, LC (lapatinib), HC (trastuzumab), PEC (pertuzumab), LHC (lapatinib and trastuzumab), and PEHC (pertuzumab and trastuzumab). The main outcomes were overall survival and response rates. We also examined side effects of rash, LVEF (left ventricular ejection fraction), fatigue, and gastrointestinal disorders, and performed subgroup analysis for the different treatment regimens in metastatic or advanced breast cancer.

**Results:**

We identified 25 articles of 21 trials, with data for 11,276 participants. T-DM1 and PEHC were more efficient drug regimens with regard to overall survival as compared with LHC, LC, HC and PEC. The incidence of treatment-related rash occurs more frequently in the patients who received LC treatment regimen than PEHC and T-DM1 and HC. In subgroup analysis, T-DM1 was associated with increased overall survival as compared with LC and HC. PEHC was associated with increased overall response as compared with LC, HC, and NST.

**Conclusions:**

Overall, the regimen of T-DM1 as well as pertuzumab in combination with trastuzumab and docetaxel is efficacious with fewer side effects as compared with other regimens, especially for advanced HER2+ breast cancer.

**Impact:**

This study suggests that both T-DM1 and PEHC therapy are potentially and equally useful treatments for HER2+ breast cancer.

## Introduction

Breast cancer, with more than 1 million new cases confirmed per year in the world[1], is the most frequently diagnosed cancer and the leading cause of cancer death in females worldwide. In 2008, breast cancer accounted for 23% (1.38 million) of all new cancer cases and 14% (458,400) of all cancer deaths [1–3]. Amplification of the human epidermal growth factor receptor 2 (HER2) gene occurs in 10%-35% of human breast cancers, and correlates with a more aggressive phenotype and poorer prognosis [4–7]. With regard to the management of HER2-positive breast cancer, trastuzumab [8–11], pertuzumab [12, 13], lapatinib [14, 15] are approved as standard care for inhibiting HER2 activity in the treatment of HER2-positive breast cancer [16], increasing the incidence of progression-free survival (PFS), overall survival (OS) and overall response rate (ORR) compared with chemotherapy alone. The TDM4450g trial reported that trastuzumab emtansine provides a better median PFS, by an increase of 5 months, compared to trastuzumab in combination with docetaxel in HER2-positive metastatic breast cancer [17]. The targeting of HER2 with more than one agent is better than use of a single agent in a first/second-line setting [9, 12, 18–20]. According to the CLEOPATRA study, HER2-positive breast cancer patients received a regimen of combining pertuzumab with trastuzumab and docetaxel, and demonstrated a significantly improvement in overall survival compared with individuals who received a regimen of trastuzumab in combination with placebo and docetaxel [12, 21]. In addition to the CLEOPATRA study, by far there have been only two randomized clinical trials of combination treatments including more than one of the above HER2-targeted drugs to treat HER2-positive breast cancer patients[22, 23]. Furthermore, no randomized clinical trial has compared a lapatinib-containing regimen directly with pertuzumab-containing or T-DM1-containing regimen, so there is a need for indirect meta-analysis to evaluate these different HER2-targeted therapies. One of prior meta-analyses did not stress on the HER2-targeted therapy [24]. The other meta-analysis did not include the HER2-targeted agents of T-DM1 and pertuzumab in the study [25].

Network meta-analysis is one of standard methods for systematic review and meta-analysis [26–37]. Such analysis more comprehensively synthesizes direct and indirect evidence to evaluate data, compared to traditional meta-analysis, which just employs direct data to demonstrate results [38, 39]. More importantly, we can isolate a relatively better treatment among several similar therapies with this network meta-analysis.

To identify relatively a better HER2-targeted treatment regimen among trastuzumab, pertuzumab, T-DM1, lapatinib in combination with standard treatment (chemotherapy or hormone therapy or endocrine therapy without HER2-targeted drugs) in HER2-positive breast cancer, we performed a comprehensive systematic network meta-analysis of HER2-targeted agents combined with standard treatment for HER2+ breast cancer and evaluated the relative merits of the different regimens. We compared overall survival rate (OSR) and overall response rate (ORR) as well as side effects for these treatments.

## Materials and Methods

### Definition of HER2-targeted therapy

Suitable HER2-targeted agents were identified through the following registries (http://www.clinicaltrials.gov;http://www.cancer.gov/search/clinical_trials/search_clinicaltrialsadvanced.aspx;http://www.who.int/trialsearch). HER2-targeted combination treatment was defined as single- or multi-targeted HER2 agents (e.g. trastuzumab, pertuzumab, lapatinib, and trastuzumab-MCC-DM1) combined with standard treatment (chemotherapy or hormone therapy, e.g. like taxane, anastrozole, letrozole, anthracycline without the addition of any HER2-targeted agents).

### Trial criteria

Prospective clinical phase II or III randomized controlled trials (RCTs) of treatment were entered into this network analysis to compare the efficacy and safety of different HER2-targeted treatment regimens in which HER2-targeted agents were combined with standard treatment in any-line HER2+ breast cancer without consideration whether the ages of these patients were or were not over 70 years of age. Trials that compared the efficacy of different HER2+ therapy dosing schedules, compared the efficacy of different administration order (sequentially or concurrently) or administration approach (orally or intravenously), evaluated HER2-targeted vaccines or reported only quality-of-life measures or pharmacokinetic outcomes were excluded.

### Search strategy

We searched for clinical trials published in English in MEDLINE via PubMed and ClinicalTrials.gov (http://www.clinicaltrials.gov) with the key words “trastuzumab”, “Herceptin”, “lapatinib”, “Tykerb”, “pertuzumab”, “Omnitarg” and the exploded MeSH term “breast neoplasms” and search line [(breast or mammary) and (cancer* and tumour* and tumor* or neoplas* or carcinoma)] [25]. The last search was on March 1, 2014. The PubMed search was restricted to RCTs and humans. We searched for additional studies in the reference lists of most primary studies and original reviews comparing regimens for HER2+ breast cancer.

Two reviewers (Qiuyan Yu and Zhenli Zhu) independently assessed the titles and abstracts of retrieved articles to determine trial inclusion. Any discrepancies were resolved by a third researcher (Ke Li). The full-text manuscripts of potential trials were reviewed.

### Data extraction and quality assessment

Two reviewers (Qiuyan Yu and Zhenli Zhu) extracted data on the trial design, patient eligibility, baseline patient characteristics, dosing regimens, line of treatment, method of HER2+ identification, duration of follow-up and risk of bias of trials. Disagreements in data extraction were resolved by discussion or by a third researcher. If trial results were reported in multiple publications, we extracted the most recently reported endpoints. The risk of bias of trials was assessed as recommended by the Cochrane Collaboration [40].

The main outcomes were OSR (overall survival rate) and ORR (overall response rate) according to the Response Evaluation Criteria in Solid Tumors (RECIST) or the WHO criteria. Secondary outcomes were the side effects rash, left ventricle ejection fraction (LVEF, 10–50%), fatigue, and gastrointestinal disorders (diarrhea, nausea, vomiting) according to the National Cancer Institute (NCI) Common Toxicity Criteria version 2 or Common Terminology Criteria for Adverse Events (CTCAE) version 3. For OSR, ORR and side effects, we extracted or derived the number of patients with outcome of interest (numerator) and total number of patients in the treatment arm (denominator). OSR was defined as the proportion of survivors from the time of randomization to the recently reported endpoint for any casualty. The intent-to-treat analysis was used for the evaluation of the OSR, ORR and side-effects.

### Treatment categories

We investigated the following 6 regimens: T-DM1 (trastuzumab emtansine), LC (lapatinib), HC (trastuzumab), PEC (pertuzumab), LHC (lapatinib and trastuzumab), and PEHC (pertuzumab and trastuzumab). Other therapies such as anthracycline, taxane, capecitabine, anastrozole, letrozole, vinorelbine without the addition of any-targeted agents were grouped as naïve standard treatments (NST) [24].

### Statistical analyses

OSR and ORR were analyzed by the total number of randomly assigned participants as the denominator [41]. For OSR, if we could not extract survival data, but death data was reported, we used this data to inversely calculate survival. For side effects, if only percentages were reported, we estimated the nearest whole number of events instead of the actual number of incidents [42].

For every direct comparison of HER2-targeted treatment regimens, we first synthesized data, which was extracted from the randomized clinical trials with the same HER2-targeted treatment comparisons, to compare the efficacy and safety of these trials with each other with a random-effects model [41–44]. Both odds ratios (ORs) and 95% confidence intervals (95% CIs) were calculated for dichotomous outcomes. The I^2^ statistic as well as forest plots were computed to determine whether there was statistical heterogeneity, with low heterogeneity defined as an I^2^ value <25%, and high heterogeneity defined as an I^2^ value >50% [45].

A network meta-analysis was employed to synthesize direct and indirect treatment regimens to assess the therapeutic effect between all the HER2-targeted combination treatments, and was used to rank these results graphically [38, 39, 46]. The Markov Chain Monte Carlo method was used to estimate these regimens [41, 42, 46]. And judge whether the residual deviance was similar with the number of data points with the approach of the posterior mean [42, 47]. For every treatment regimen, we calculated the probability of its efficacy and safety and ranked treatments by rank-grams [39].

In estimating the consistency between direct and indirect evidence, the data from both sources was checked by the Bucher method to determine whether it was so similar enough that we could integrate the direct and the indirect evidence together [42, 47–49]. That is, we calculated the difference between direct and indirect evidence in all closed loops in the network. Once one closed loop was identified with a 95% CI excluding 0, we could confirm clearly the existing differences between the direct and indirect evidence [41, 50].

We performed a sensitivity analysis using a fixed-effects model by repeating the main computations. As well, we performed a subgroup analysis using a random-effects model for advanced HER2+ breast cancer.

Analysis involved use of STATA 12.0 (pair-wise meta-analysis and I^2^ calculation), R 3.0.2 (estimation of consistency between direct and indirect evidence, rank-grams, and SUCRA graphs), and WinBUGS 1.4.2 (multiple-treatments of meta-analysis models).

## Results

### Eligible trials

**Fig 1:**
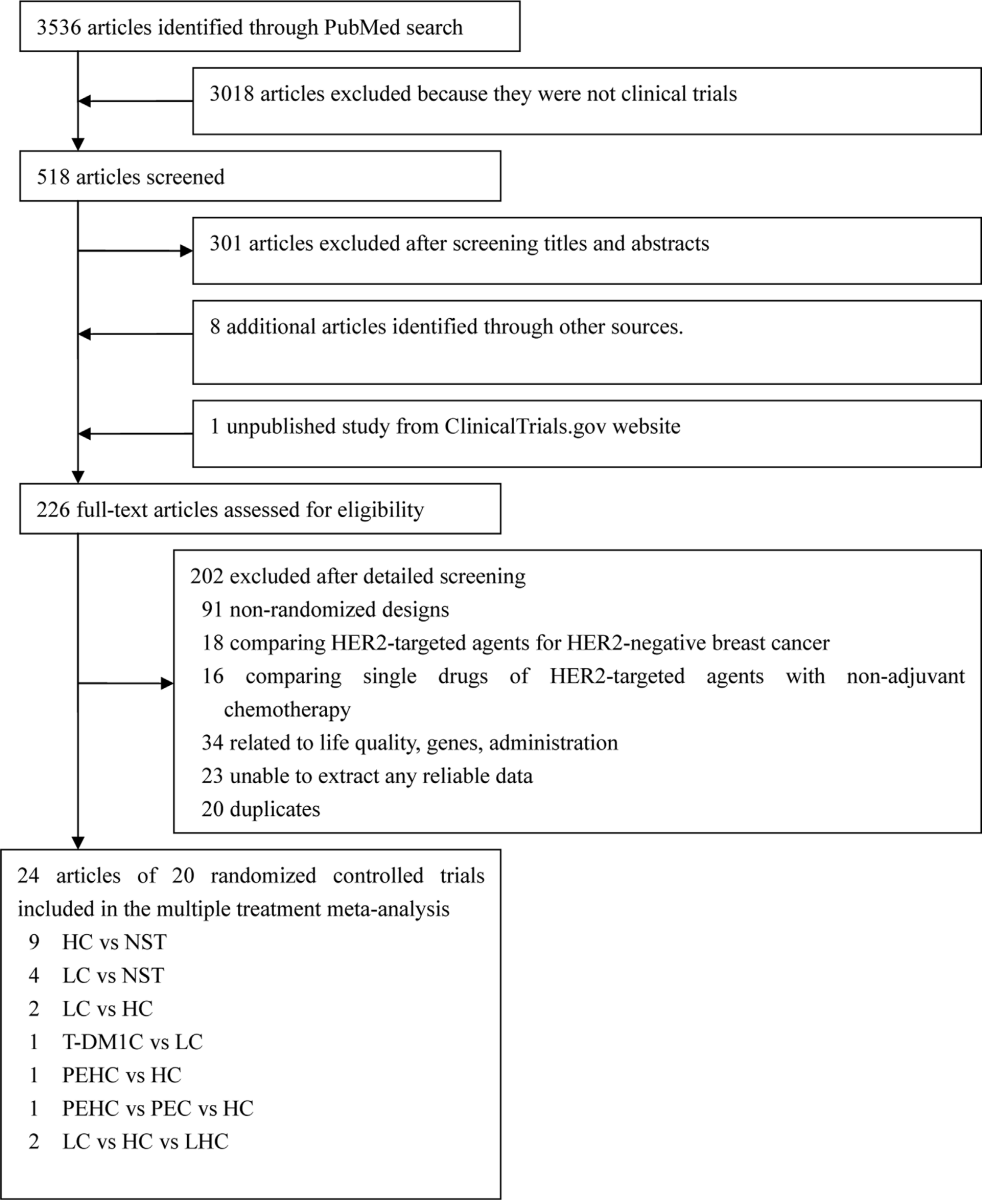
Flow of articles in the study. 21 randomized trials corresponding to 43 groups because 3 were three-group studies.

Abstract and titles of 575 articles were identified for first review employing the search strategies (S1 Table) as described in Fig 1. After objectively reading the titles and abstracts, 343 articles were excluded because they were not randomized controlled trials, and 121 unrelated articles were excluded, 8 additional articles identified through other sources and 1 unpublished study from ClinicalTrials.gov website were included, resulting in 120 full-texts evaluating the effect of HER2-targeted drugs in treatment of breast cancer. Of these publications, 69 articles were excluded because 13 of them provided deficient or inaccessible information and the rest were excluded because they compared HER2-targeted agents for HER2-negative or triple-negative breast cancer, and 19 articles were excluded because they focused on single drug comparison without combination with standard chemotherapy or concentrated on different administration order or approach. 7 articles were excluded because they reported end-points about life-quality, genes and cost-efficacy. In the end, 25 articles of 21 trials were included in our network meta-analysis (Fig 1) and analyzed for the following HER2-targeted regimens: T-DM1, LC, HC, PEC, PEHC, LHC and NST (S2 Table). Most trials (n = 18 [85%]) were two-group studies and the remainder were three-group trials [12, 14, 15, 17, 18, 22, 23, 51–67]. Overall, 11,276 patients were randomly assigned to 1 of the 6 HER2-targeted treatments or to naïve standard treatment (NST). The mean sample size was 250 patients per group (range 26–1097 patients). The mean duration of studies was 31 months (2 studies, <10 months; 15, 10–50 months, and 4, >50 months). Supplementary unpublished information was obtained from other reviews and trial investigators. All included studies recruited patients described as grade 2-plus with positive on immunohistochemistry (IHC2+), IHC3+, or positive on fluorescence *in situ* histochemistry for HER2.

The number of adverse events was inconsistently reported. More reports counted the events of gastrointestinal disorders as grades 3-plus rather than grades 1–4. So, we used the number of gastrointestinal disorder events as grades 3-plus [42]. However, for the side effects of rash and fatigue, we used the number of all grades to evaluate safety. The number of LVEF cases was imputed as the proportion between 10% and 50%. We found 5,927 cases of overall survival, 2,759 of overall response, 1,334 of rash, 1,918 of fatigue, 397 of diarrhea, 288 of nausea, 214 of vomiting, and 654 of abnormal LVEF (10%-50%).

The overall quality of studies was rated moderate (S1 and S2 Figs). Some studies did not record details about concealment. We judged studies which reported concealment by a central office or an interactive third-party telephone via an interactive voice response system or web-based randomizations via an interactive web-based response system as having no bias.

### Traditional meta-analysis

We performed a series of conventional meta-analyses to summarize the same classes of treatment regimens. OSR was better with T-DM1 than LC or with PEHC than HC (S3 Table). ORR was better with PEHC than PEC and HC or with T-DM1 than LC. We found no significant heterogeneity with I^2^ >50%. In side-effect analysis, we found significant heterogeneity in the meta-analysis of rash for the comparisons LC versus NST, LVEF (10%-50%) for HC versus NST, fatigue for HC versus NST, and diarrhea for LC versus HC (S3 Table). For the meta-analysis of diarrhea, we found I^2^ > 75% for the comparisons LC versus HC, which represented the difference of treatment-related diarrhea between LC and LC have statistical significance.

### Network meta-analysis

**Fig 2:**
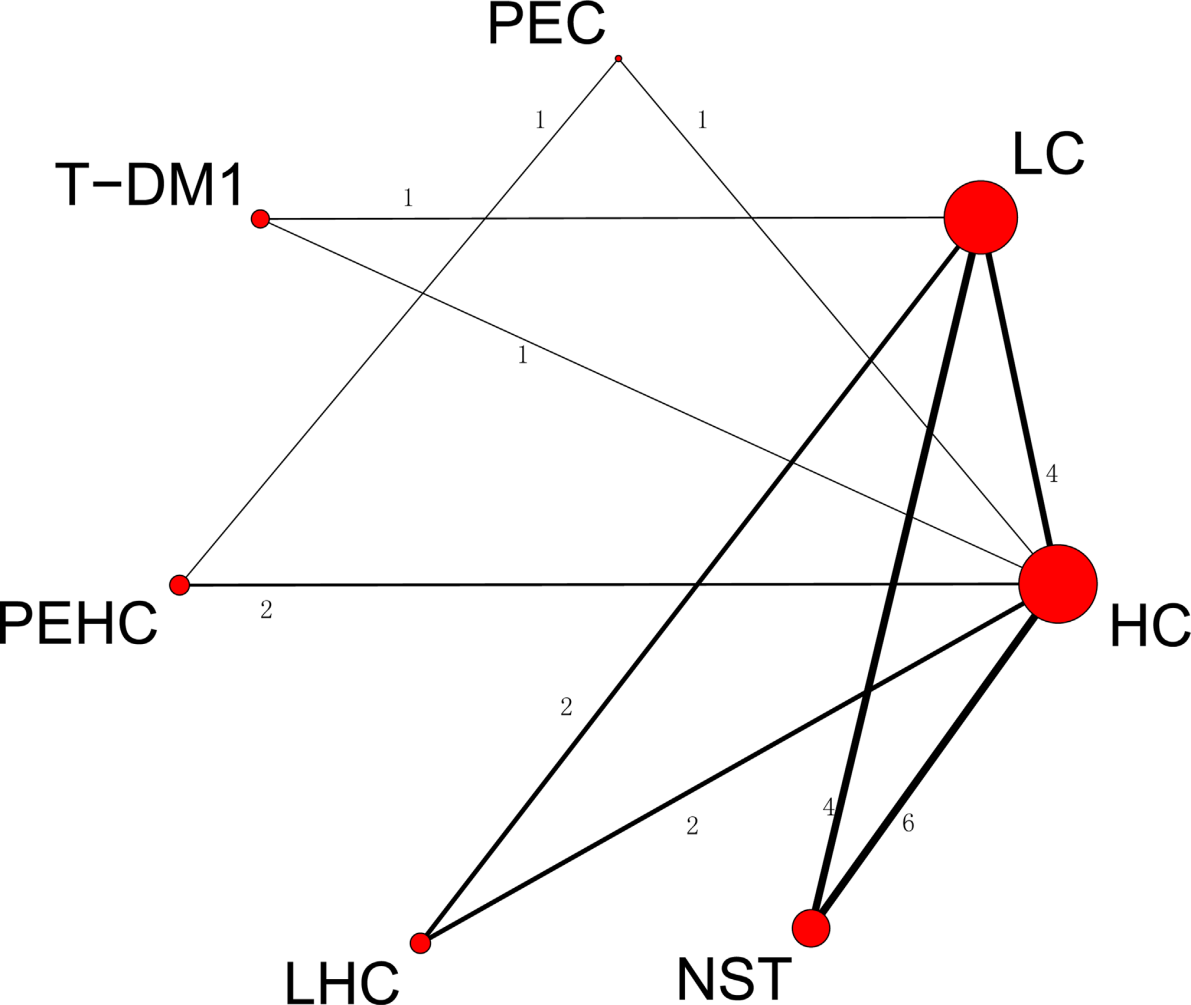
Network of comparisons for overall response rate. The size of the nodes corresponds to the number of treatment study trials. Direct comparison treatments are linked with a line noting the number of trials. The thickness of the line corresponds to the number of trials that studied this comparison. Node: T-DM1; LC, lapatinib; HC, trastuzumab; NST, naïve standard treatments; PEHC, pertuzumab and trastuzumab; PEC, pertuzumab; LHC, lapatinib and trastuzumab.

The ORR and OSR of the 7 treatment regimens for are in Fig 2 and S3 Fig, respectively. The size of nodes related to treatment with T-DM1, LC, HC, NST, LHC, PEHC and PEC corresponded to the number of randomized participants (sample size) that studied the treatments. Treatments that were directly compared are linked with a line and the number of trials. For example, the direct comparison between HC versus NST treatments is linked with a line noting 6 trials. The thickness of the line corresponds to the numbers of trials that studied this comparison. Of the 21 pair-wise comparisons between the 7 treatment regimens, 17 trials directly studied efficacy for OSR and ORR and 20 trials for safety.

In head-to-head comparisons, for OSR, the T-DM1 regimen was more effective than LC (OR 0.60 [95% CI 0.39, 0.94]) (Table 1). For ORR, the T-DM1 regimen was more effective than LC (OR 0.59 [0.40, 0.85]) and PEC (OR 0.45 [0.18, 0.91]), and the PEHC regimen was more effective than LC (OR 2.06 [1.29, 3.16]), HC (OR 1.85 [1.23, 2.67]), or PEC (OR 3.04 [1.48, 5.60]) (Table 2). Their cumulative SUCRA ranking curves for overall survival rate and overall response rate were shown in S5 Fig.

**Table 1:** Network meta-analysis comparison of the efficacy of the 5 HER2-targeted treatment regimens for overall survival rate (OSR).

**T-DM1**	**0.61 (0.39,0.94)** *	0.60 (0.33,1.07)	**0.44 (0.24,0.73)** *	0.88 (0.40,1.76)
	**LC**	0.99 (0.71,1.35)	**0.71 (0.52,0.94)** *	1.43 (0.76,2.65)
		**HC**	**0.73 (0.59,0.89)** *	1.44 (0.90,2.28)
	**OSR**		**NST**	**2.01 (1.17,3.39)** *
				**PEHC**
T-DM1	0.61 (0.39,0.94) *	0.60 (0.33,1.07)	0.44 (0.24,0.73)*	**0.88 (0.40,1.76)**
	LC	0.99 (0.71,1.35)	0.71 (0.52,0.94) *	**1.43 (0.76,2.65)**
		HC	0.73 (0.59,0.89) *	**1.44 (0.90,2.28)**
	OSR		NST	**2.01 (1.17,3.39)** *
				**PEHC**

* The difference between A and B has statistical significance with its confidence interval without 1.

**Table 2:** Network meta-analysis comparison of the efficacy of the the 7 HER2-targeted treatment regimens for overall response rate (ORR).

**T-DM1**	**0.59 (0.40,0.85)** *	0.66 (0.42,1.02)	**0.27 (0.17,0.43)** *	**0.45 (0.18,0.91)** *	1.23 (0.66,2.11)	1.03 (0.53,1.76)
	**LC**	1.12 (0.97,1.42)	**0.45 (0.36,0.58)** *	0.75 (0.35,1.38)	**2.06 (1.29,3.16)** *	1.72 (0.99,2.81)
		**HC**	**0.41 (0.33,0.52)** *	0.67 (0.33,1.23)	**1.85 (1.23,2.66)** *	1.54 (0.89,2.42)
			**NST**	1.64 (0.77,3.01)	**4.53 (2.79,6.94)** *	**3.80 (1.13,6.40)** *
	**ORR**			**PEC**	**3.04 (1.48,5.60)** *	**2.59 (1.25, 5.26)** *
					**PEHC**	0.86 (0.45, 1.55)
						**LHC**

* The difference between A and B has statistical significance with its confidence interval without 1

The results for safety for head-to-head comparisons are in S4 Table. The LHC regimen showed greater association with rash than the T-DM1, HC, PEHC and NST regimens, and the PEHC regimen was less associated with rash than the LC regimen. The LC regimen was less associated with diarrhea than the NST and HC regimens. We found no significant effects for LVEF, fatigue, nausea and vomiting, perhaps because of the small sample size. Most loops were consistent. Only for rash for LC versus HC versus NST showed low inconsistency (S4C Fig), which implies that the direct estimate of the summary effect should differ from the indirect estimate according to the loop plots.

S6 Table represented 5 regimens ordered by their probability of being the best in terms of OSR as well as all HER2-targeted treatment regimens ordered by their probability of being the best in terms of ORR, showing the separate contributions of percentage to the overall scores of efficacy are shown.

We performed a sensitivity analysis and found that random- and fixed-effects models produced the similar results (S5, S7 and S8 Tables). Many meta-analyses have focused on advanced or metastasized breast cancer, so we performed a subgroup analysis of HER2+ advanced breast cancer with the T-DM1, LC, HC, NST, PEHC regimens (S9 Table). For OSR, the T-DM1 regimen was more effective than LC, HC and NST. For ORR, the T-DM1 regimen was more effective than LC and NST, and the PEHC regimen was more effective than LC, HC and NST. Rash was more associated with LC than T-DM1 and more associated with LC than HC, and NST. The regimens of T-DM1 and PEHC seemed to be better for HER2-targeted therapy for advanced or metastatic breast cancer with HER2-overexpression (S10 Table).

## Discussion

In our network meta-analysis, treatment with T-DM1 or pertuzumab in combination with trastuzumab and docetaxel, outperformed other treatment regimens, and the treatment effects were indistinguishable in magnitude. We found no difference in outcomes among the LC, HC, and LHC regimens for treatment of HER2+ breast cancer. In terms of side effects, rash was more associated with the LHC regimen than PEHC, HC, and T-DM1 regimens, and less associated with the PEHC than LC regimen. The PEC regimen did not differ from other 6 regimens perhaps because of limited sample size. Adding pertuzumab to standard treatment was previously found to be associated with a significant risk of rash [68]. Furthermore, risk of abnormal LVEF was greater with adding trastuzumab to standard treatment than naïve standard treatment [69]. Overall, no two HER2-targeted treatment regimens were significantly different with regard to the remaining side effects.

With 7 treatment regimens for HER2+ breast cancer, direct comparisons are limited clearly by the small number of studies evaluating a specific pair of treatment regimens. Network meta-analysis resolved this problem using indirect comparisons and distinguishes the most effective and safest treatment. Nonetheless, we found no meaningful data for the side effects of fatigue, diarrhea, vomiting and nausea.

A mixed model is as the most appropriate for multiple-treatment meta-analysis [38, 48]. Except for a heterogeneity >75% for the LC versus HC regimens for diarrhea, we found little heterogeneity among all peer-comparisons. The categories of HER2-targeted treatment might be the first attempt to be used to compare direct and indirect data with the network approaches for HER2-targeted treatment. Some network or conventional analyses have been published for advanced breast cancer, but they either just focused on targeted therapy without stressing on the HER2-targeted therapy or did not use meta-analysis for multiple-treatments [24, 25].

Our evidence supports adding HER2-targeted agents to standard treatments for HER2+ advanced or metastatic breast cancer, but the optimal duration of ErbB-family receptor inhibitors added to standard treatment is not clear. In our network meta-analysis and pair-wise meta-analysis, OSR and ORR was associated more with the T-DM1 than LC regimen [70], more with HC and LC regimens than naive standard treatment (NST) [71], and more with the LHC regimen than LC and HC regimens, although linked to rash and gastrointestinal disorders. In the subgroup analysis, because of the limited of sample sizes, we included T-DM1, LC, HC, and PEHC as well as NST for analysis of OSR and all 7 treatment regimens for analysis of ORR. The best treatment was T-DM1 for OSR and PEHC for ORR. In our network analysis, we found T-DM1 and PEHC regimens were potentially and equally as ideal HER2-targeted treatment regimens for treating HER2+ breast cancer.

Our study has some disadvantages. First, the network could not be expanded to unpublished trials and extracted published data rather than individual patient information [42, 72]. Secondly, we chose the suitable trials without consideration of whether the trial was first-line or not because of lack of sufficient trials. Although the accuracy of the results might be misleading to some extent, we performed a subgroup analysis for first/second-line HER2+ advanced or metastatic breast cancer and got more reliable results. In addition, some trials were open-label and the concealment was not detailed clearly; thus, the validity of the results might be underestimated[41]. Third, in any meta-analysis, selection bias and publication bias cannot be avoided. In subgroup analysis, we did not estimate the effect on early HER2+ breast cancer because only 4 trials were available on this topic, which is insufficient for constructing a closed loop. Finally, our study did not stress on high- and low-dosing HER2-targeted agents combined with standard treatments. If we detail such treatment regimens like that, these regimens can not be connected with each other in a closed loop. In the future, with the increasing number of clinical trials for HER2+ breast cancer, we hope to provide details for HER2-targeted agents added to concrete standard treatments to provide meaningful data to clinicians and researchers in this field.

## Conclusions

In conclusion, our study supports that treatment with T-DM1 as well as pertuzumab in combination with trastuzumab and docetaxel, currently is the best targeted drug regimen for HER2+ breast cancer in terms of overall survival rate (OSR) and overall response rate (ORR) and with moderate toxicity, especially for HER2+ advanced or metastatic breast cancer. Naive standard treatment (NST) without the HER2-targeted agents showed poor efficacy, but the least toxicity. The suggested hierarchies for the 7 treatment regimens should provide information for clinicians to choose a suitable targeted therapy for HER2+ breast cancer. We hope that more and more randomized trials, consisting of more than one HER2-targeted agent in combination with chemotherapy/hormone therapy in HER2-positive breast cancer such as the comparable randomized trials that T-DM1+/- pertuzumab versus standard treatment with or without T-MD1+/- pertuzumab, can be increased to represent various and efficacious treatment regimens.

## Supporting Information

S1 Fig Assessment of bias in included trials. (TIF)

S2 Fig Risk of bias for each Cochrane component. Review authors’ judgments (low, unclear, high) for each risk of bias item presented as percentages across 19 included studies, one study was from the ClinicalTrials.gov website without bias description.
(TIF)

S3 Fig Network of comparisons for overall survival rate (OSR). The size of the nodes corresponds to the number of trials that studied the treatment. The directly comparable treatments are linked with a line. The thickness of the line corresponds to the number of trials that studied this comparison. T-DM1C, T-DM1; LC, lapatinib; HC, trastuzumab; NST, naïve standard treatments; PEHC, pertuzumab and trastuzumab; PEC, pertuzumab; LHC, lapatinib and trastuzumab.
(TIF)

S4 Fig Inconsistency in efficacy for overall survival rate(A), overall response rate(B) and induction of rash(C). Inconsistency estimated as the difference between direct and indirect estimate (called inconsistent factor; IF) and the corresponding 95% confidence interval (95% CI) for the IF in each closed loop. The forest plots show all closed triangular loops (loops formed by 3 treatments) in each outcome network. Inconsistent loops present inconsistent loops per network (maximum 9% of the loops), which can be attributed to chance.
(TIF)

S5 Fig Cumulative SUCRA ranking curves for overall survival rate(A) and overall response rate(B). (TIF)

S1 PRISMA Checklist (DOC)

S1 Table Search strategy in PubMed. (DOC)

S2 Table Characteristics of included trials. (DOC)

S3 Table Meta-analysis of direct comparisons for efficacy and safety. (DOC)

S4 Table Safety of the 7 HER2-targeted treatment regimens in network meta-analysis. (DOC)

S5 Table Model fit in random-effects model and fixed-effects model. (DOC)

S6 Table Ranking for efficacy and safety with random-effects models. (DOC)

S7 Table Sensitivity analysis with fixed-effect models on the efficacy and safety of 7 regimens in network meta-analysis. (DOC)

S8 Table Ranking for efficacy and safety using fixed-effects models. (DOC)

S9 Table Subgroup analysis with random-effects models on efficacy and safety of HER2+ advance or metastatic breast cancer (OR 95%CI). (DOC)

S10 Table Ranking for efficacy and safety with fixed-effects models in subgroup analysis. (DOC)
