# Supplementary figures and images for: Efficacy and Safety of HER2-Targeted Agents for Breast Cancer with HER2-Overexpression: A Network Meta-Analysis
Source: PLoS One. 2015 May 20;10(5):e0127404. doi: 10.1371/journal.pone.0127404 (PMC4439018; doi:10.1371/journal.pone.0127404)

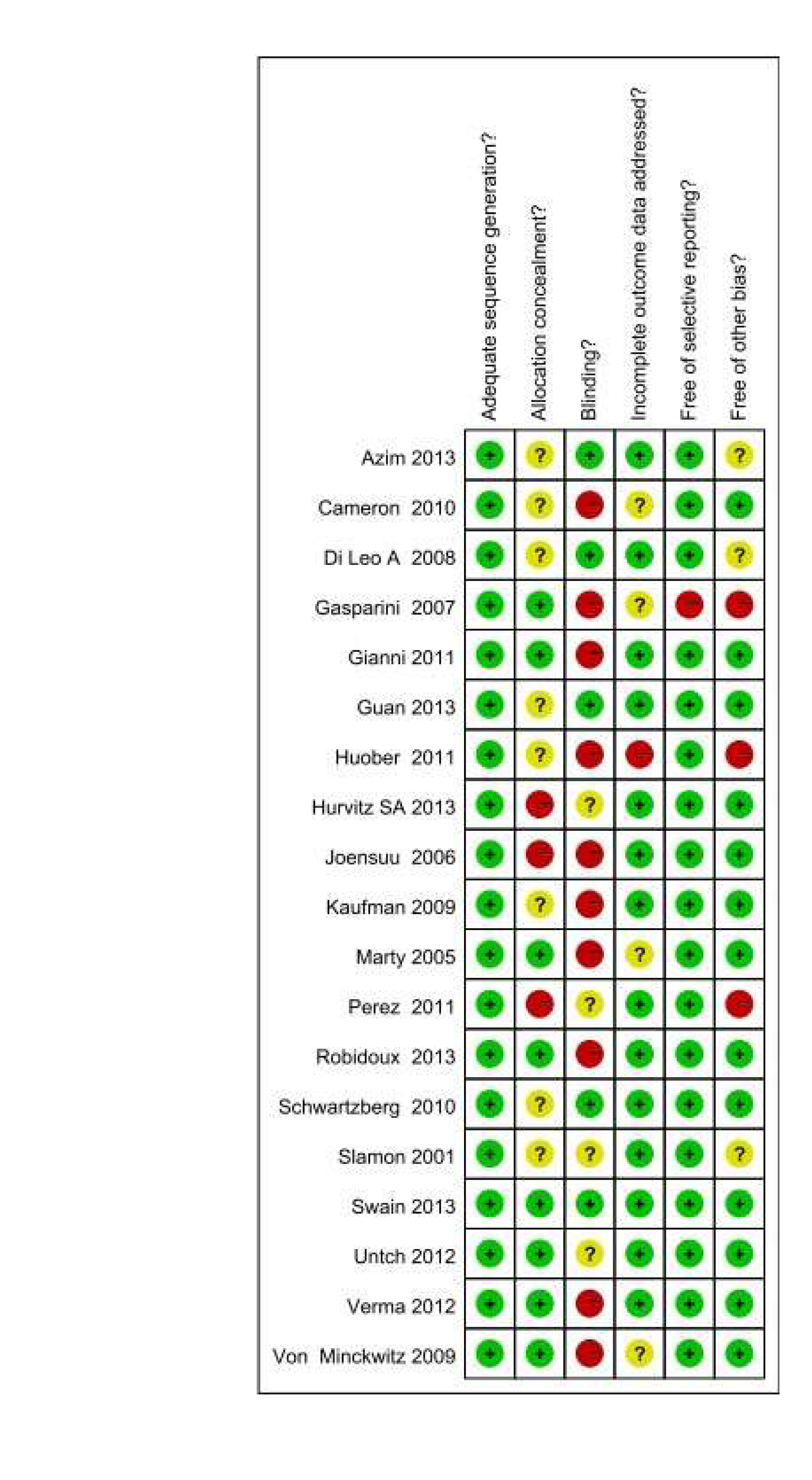

Supplement: S1 Fig — (TIF) [file pone.0127404.s001.tif]

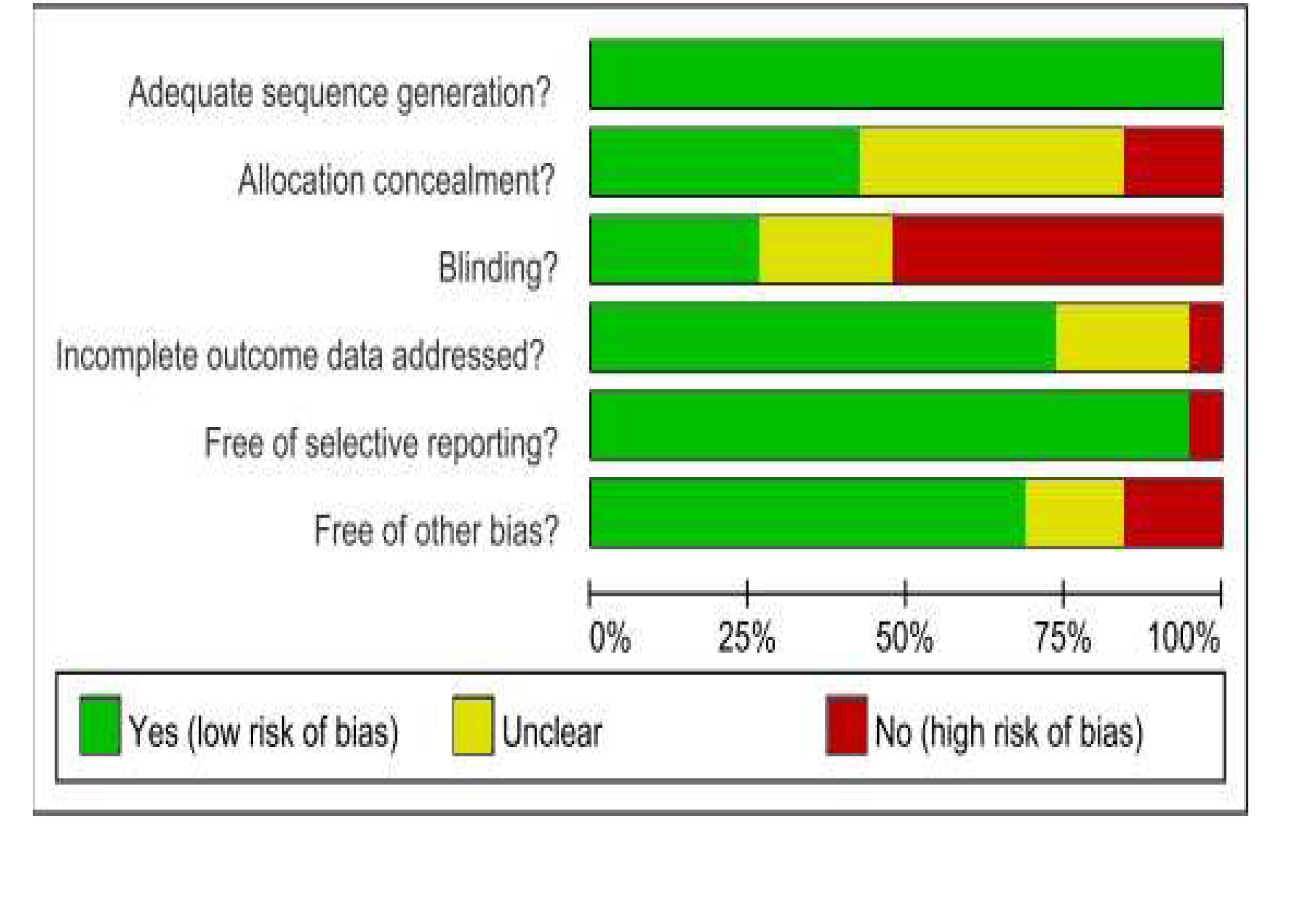

Supplement: S2 Fig — Review authors’ judgments (low, unclear, high) for each risk of bias item presented as percentages across 19 included studies, one study was from the ClinicalTrials.gov website without bias description. (TIF) [file pone.0127404.s002.tif]

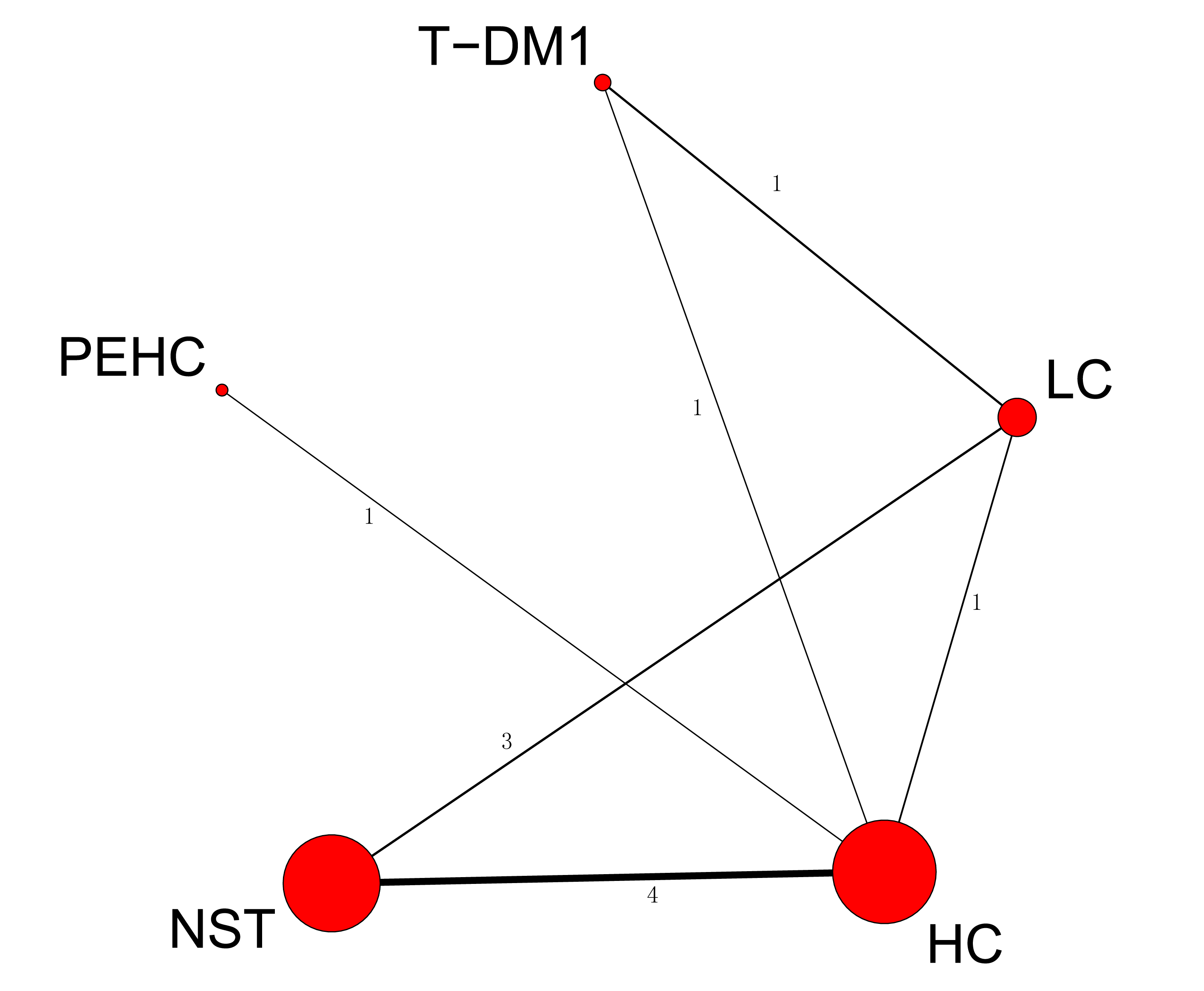

Supplement: S3 Fig — The size of the nodes corresponds to the number of trials that studied the treatment. The directly comparable treatments are linked with a line. The thickness of the line corresponds to the number of trials that studied this comparison. T-DM1C, T-DM1; LC, lapatinib; HC, trastuzumab; NST, naïve standard treatments; PEHC, pertuzumab and trastuzumab; PEC, pertuzumab; LHC, lapatinib and trastuzumab. (TIF) [file pone.0127404.s003.tif]

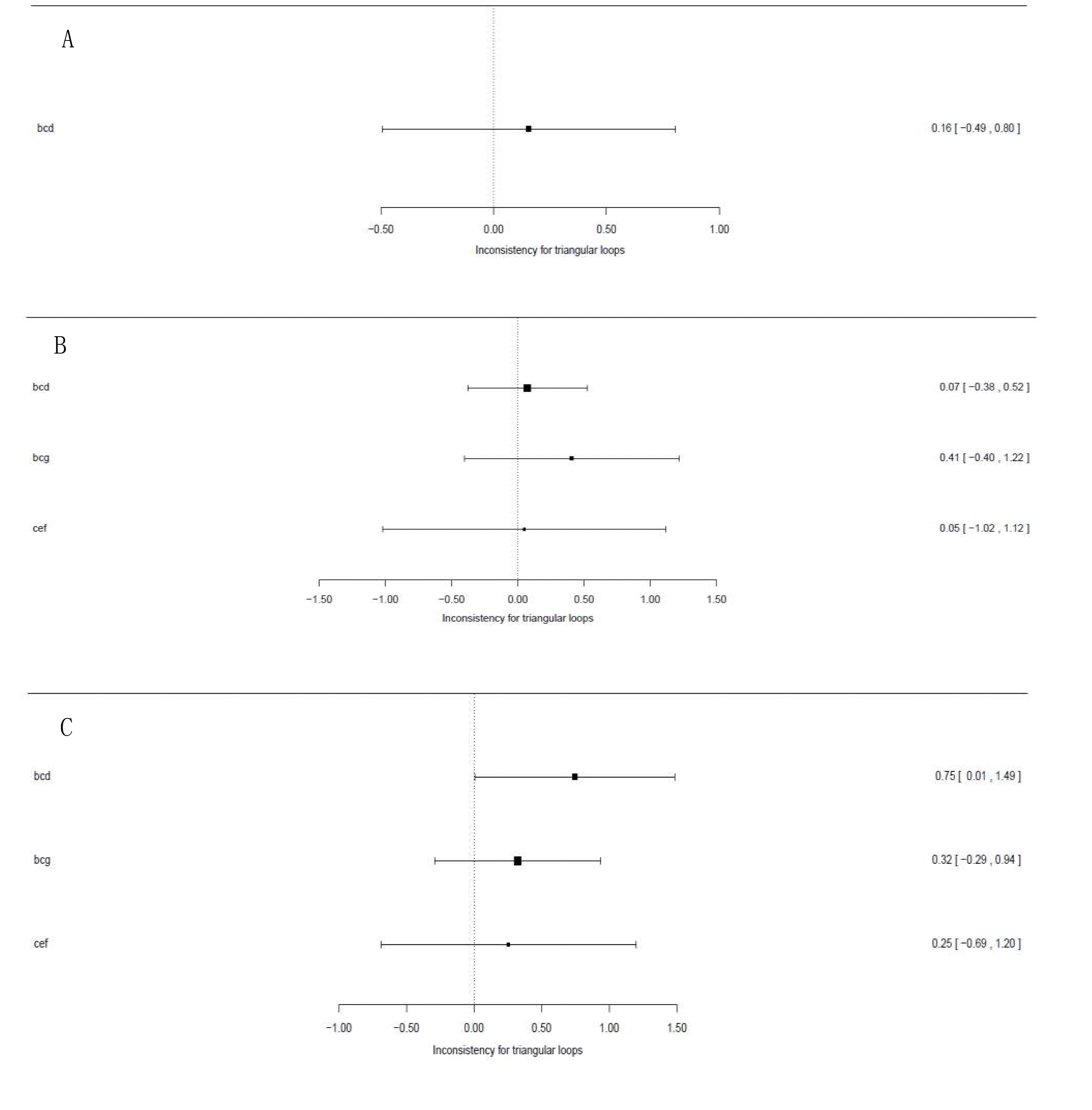

Supplement: S4 Fig — Inconsistency estimated as the difference between direct and indirect estimate (called inconsistent factor; IF) and the corresponding 95% confidence interval (95% CI) for the IF in each closed loop. The forest plots show all closed triangular loops (loops formed by 3 treatments) in each outcome network. Inconsistent loops present inconsistent loops per network (maximum 9% of the loops), which can be attributed to chance. (TIF) [file pone.0127404.s004.tif]

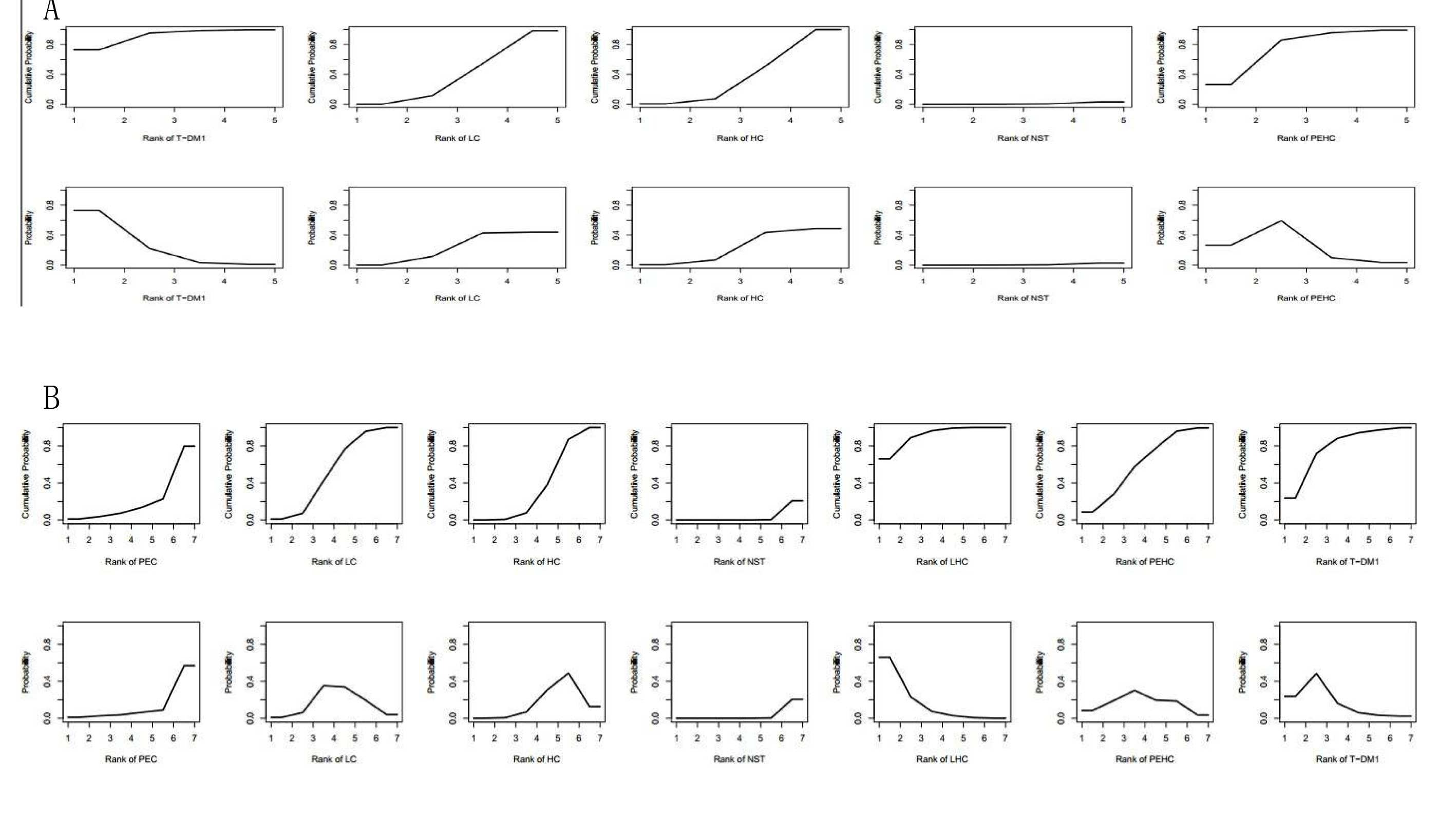

Supplement: S5 Fig — (TIF) [file pone.0127404.s005.tif]
