# Supplementary material for: Efficacy and Safety of HER2-Targeted Agents for Breast Cancer with HER2-Overexpression: A Network Meta-Analysis
Source: PLoS One. 2015 May 20;10(5):e0127404. doi: 10.1371/journal.pone.0127404 (PMC4439018; doi:10.1371/journal.pone.0127404)
Supplement: S1 PRISMA Checklist — (DOC) [file pone.0127404.s006.doc]

| **Section/topic** | **#** | **Checklist item** | **Reported on page #** |
| --- | --- | --- | --- |
| **TITLE** | | |  |
| Title | 1 | **Efficacy and safety of HER2-targeted agents for breast cancer with HER2-overexpression: a network meta-analysis** | #1 |
| **ABSTRACT** | | |  |
| Structured summary | 2 | **Background**: Clinical trials of human epidermal growth factor receptor 2 (HER2)-targeted agents added to standard treatment have been efficacious for HER2-positive (HER2+) breast cancer. No meta-analysis has evaluated HER2-targeted therapy and ranked the targeted treatments. We performed a network meta-analysis of both direct and indirect comparisons to evaluate the effect of adding HER2-targeted agents to standard treatment and examined side effects.  **Methods**: We performed a Bayesian-framework network meta-analysis of randomized controlled trials to compare 6 HER2-targeted treatment regimens and 1 naïve standard treatment (NST, without any-targeted drugs) in targeted treatment of HER2+ breast cancer in adults. These treatment regimens were T-DM1C (trastuzumab emtansine), LC (lapatinib), HC (trastuzumab), PEC (pertuzumab), LHC (lapatinib and trastuzumab), and PEHC (pertuzumab and trastuzumab). The main outcomes were overall survival and response rates. We also examined side effects of rash, LVEF, fatigue, gastrointestinal disorders. We performed subgroup analysis for the different treatment regimens in metastatic or advanced breast cancer.  **Results**: We identified 24 articles of 20 trials, with data for 11,139 participants. T-DM1C and PEHC represented more efficient classes of drugs increasing the chance of survival as compared with LHC, LC, HC and PEC. The development of rash one treatment-related side effect was more liable to the patients who received LC treatment regimen than PEHC and T-DM1C and HC. In subgroup analysis, T-DM1C was associated with increased overall survival rate as compared with LC and HC. PEHC was associated with increased overall response rate as compared with LC, HC, and NST.  **Limitations:** First, the network could not be expanded to unpublished trials and extracted published data rather than individual patient information. Secondly, we chose the suitable trials without consideration whether the trial is first-line or not because of lack of enough trials. Although the accuracy of the results might be misled in some extent, we had attempted to go through a subgroup analysis for first/second-line HER2+ advanced or metastatic breast cancer and got more reliable results. In addition, some trials were open-label and the concealment was not detailed clearly; thus, the validity of the results might be underestimated**.**  **Conclusions:** Overall, T-DM1 in combination with capecitabine as well as pertuzumab in combination with trastuzumab and docetaxel might be efficacious with fewer side effects as compared with other regimens, especially for advanced HER2+ breast cancer.  **Impact**: This study suggests that T-DM1C or PEHC therapy is as probably useful treatments for HER2+ breast cancer.  No systematic review registration number. | #2 |
| **INTRODUCTION** | | |  |
| Rationale | 3 | Breast cancer, with more than 1 million new cases confirmed per year in the world, is the most frequently diagnosed cancer and the leading cause of cancer death in females worldwide. It accounted for 23% (1.38 million) of all new cancer cases and 14% (458,400) of all cancer deaths in 2008. Amplification of the human epidermal growth factor receptor 2 (HER2) genes, occupies 10%-35% of human breast cancer, is related with a more aggressive phenotype and poorer prognosis. With regard to the management of HER2-positive breast cancer, trastuzumab, pertuzumab, lapatinib are approved as the standard care to inhibit HER2 activity for HER2-positive breast cancer, apparently getting clinical benefit in progression-free survival (PFS), overall survival (OS) and overall response rate (ORR) compared with chemotherapy alone. In TDM4450g trial, trastuzumab emtansine represents better median PFS of increased 5 months than trastuzumab in combination with docetaxel in HER2-positive metastatic breast cancer. Targeting of HER2 with more than one agent is better than use of one agent only in many clinical trials in first/second-line setting. In CLEOPATRA study, HER2-positive breast cancer patients received a regimen of combining pertuzumab with trastuzumab and docetaxel demonstrated a significantly improvement in overall survival compared with individual who received a regimen of trastuzumab in combination with placebo and docetaxel. In addition of the CLEOPATRA study, by far there have been only two combination treatments of randomized clinical trial including more than one of the above HER2-targeted drugs to treat HER2-positive breast cancer patients. Furthermore, not a randomized clinical trial has compared lapatinib-contained regimen directly with pertuzumab-contained or T-DM1-contained regimen, so employing indirect meta-analysis is a need to evaluate these different HER2-targeted therapy. One meta-analysis didn’t stress on the HER2-targeted therapy. In another meta-analysis, it did not include the HER2-targeted agents of T-DM1 and pertuzumab in its research. | #3 |
| Objectives | 4 | To provide relatively a better HER2-targeted treatment regimen among trastuzumab, pertuzumab, T-DM1, lapatinib in combination with standard treatment (chemotherapy or hormone therapy or endocrine therapy without HER2-targeted drugs) in HER2-positive advanced breast cancer, it is of rationale to perform a comprehensive systematic network meta-analysis of HER2-targeted agents combined with standard treatment for HER2+ breast cancer and evaluated the relative merits of the different regimens. We compared overall survival rate (OSR) and overall response rate (ORR) as well as side effects for these treatments. | #3 |
| **METHODS** | | |  |
| Protocol and registration | 5 | No protocol and registration. |  |
| Eligibility criteria | 6 | Prospective clinical phase Ⅱor Ⅲ randomized controlled trials (RCTs) of treatment were entered into this network analysis to compare the efficacy and safety of different HER2-targeted treatment regimens that added the HER2-targeted agents to standard treatment in any-line HER2+ breast cancer without consideration whether the ages of these patients were 70 plus or not. if trials compared the efficacy of different HER2+ therapy dosing schedules, compared the efficacy of different administration order (sequentially or concurrently) or administration approach (orally or intravenously), evaluated HER2-targeted vaccines or reported only quality-of-life measures or pharmacokinetic outcomes were excluded. | #4 |
| Information sources | 7 | We searched PubMed to identify suitable studies prior to 1st March, 2014. No start data limit was applied. | #4 |
| Search | 8 | the key words “trastuzumab”, “Herceptin”, “lapatinib”, “Tykerb”, “pertuzumab”, “Omnitarg”, “neratinib”, “HKI272” and the exploded MeSH term “breast neoplasms” and search line [(breast or mammary) and (cancer* and tumour* and tumor* or neoplas* or carcinoma)]. The last search was on March 1, 2014. The PubMed search was restricted to RCTs and humans. | #4 |
| Study selection | 9 | Two reviewers (Q Yu and Z Zhu) independently assessed the titles and abstracts of retrieved articles to determine trial inclusion. Any discrepancies were resolved by a third researcher (K Li). The full-text manuscripts of potential trials were reviewed. | #4 |
| Data collection process | 10 | Two reviewers (Q Yu and Z Zhu) extracted data on the trial design, patient eligibility, baseline patient characteristics, dosing regimens, line of treatment, method of HER2+ identification, duration of follow-up and risk of bias of trials. Disagreements in data extraction were resolved by discussion or by a third researcher. If trial results were reported in multiple publications, we extracted the most recently reported endpoints. | #5 |
| Data items | 11 | The main outcomes were OSR (overall survival rate) and ORR (overall response rate) according to the Response Evaluation Criteria In Solid Tumors (RECIST) or the WHO criteria. Secondary outcomes were the side effects rash, left ventricle ejection fraction (LVEF, 10-50%), fatigue, and gastrointestinal disorders (diarrhea, nausea, vomiting) according to the National Cancer Institute (NCI) Common Toxicity Criteria version 2 or Common Terminology Criteria for Adverse Events (CTCAE) version 3. | #5 |
| Risk of bias in individual studies | 12 | The network could not be expanded to unpublished trials, which could increase the number of trials examined and obtain more accurate and reliable evidence. Moreover, we extracted published data rather than individual patient information, which provided more concrete appraisal for risk groups, but the power to detect efficacious modifications, may be limited. | #11 |
| Summary measures | 13 | For every pair-wise comparison of HER2-targeted treatment regimens, we first synthesized data from studies comparing the same regimens using a random-effects model to incorporate the assumption that different studies assessed different yet related treatment effects. We calculated odds ratios (ORs) and 95% confidence intervals (95% CIs) for dichotomous outcomes. Statistical heterogeneity was inspected mainly by the I^2^ statistic as well as forest plots. I^2^ value <25% was considered low heterogeneity and >50% high heterogeneity.  We used a network meta-analysis to combine the direct and indirect treatment regimens for assessing the effects among all the interventions and ranked results graphically. The Markow Chain Monte Carlo method was used to estimate the HER2-targeted treatment regimens with 10,000 initial iterations to burn in and the next 30,000 iterations for estimates. The fit of the model was evaluated by the posterior mean of the residual deviance and the deviance information criteria; in a good-fit model, the residual deviance approximates the number of data points.  To estimate the consistency of direct and indirect evidence, we used the Bucher method to check the assumption of whether the data for both sources were similar enough to be incorporated. Then, we calculated the difference between direct and indirect evidence in all closed loops in the network. One closed loop identified with 95% CI excluding 0 indicated discrepant between direct and indirect evidence.  We performed a sensitivity analysis using a fixed-effects model by repeating the main computations. As well, we performed a subgroup analysis using a random-effects model of early and advanced HER2+ breast cancer. Finally, we examined the results using a random-effects model with homogeneous between-trial variability. | #6 |
| Synthesis of results | 14 | **Eligible trials**  We included 24 articles of 20 trials in the network meta-analysis (**Figure 1**) and analyzed the following HER2-targeted regimens: T-DM1C, LC, HC, PEC, PEHC, LHC and NST (**Supplementary T1 Table 1**). Most trials (n=17 [85%]) were two-group studies and the remainder were three-group trials . Overall, 11,139 patients were randomly assigned to 1 of the 6 HER2-targeted treatments or to naïve standard treatment (NST). The mean sample size was 259 patients per group (range 26–1097 patients). The mean duration of studies was 31 months (2 studies, <10 months; 14, 10–50 months, and 4, > 50 months). Supplementary unpublished information was obtained from other reviews, and trial investigators. All included studies recruited patients described as grade 2 positive on immunohistochemistry (IHC2+), IHC3+, or positive on fluorescence *in situ* histochemistry.  The number of adverse events was reported inconsistently. Some reports represented gastrointestinal disorders as grades 1 to 4 and the others as ≥ 3 grade. So, we used the number of gastrointestinal disorder events graded ≥ 3 grade . However, for the side effects of rash and fatigue, we used the number of all grades to evaluate safety. The number of LVEF cases was imputed as the proportion between 10% and 50%. We found 5,927 cases of overall survival, 2,759 of overall response, 1,334 of rash, 1,918 of fatigue, 397 of diarrhea, 288 of nausea, 214 of vomiting, and 654 of abnormal LVEF (10%-50%).  The overall quality of studies was rated moderate (**Supplementary F2 Figures 1-2**). Some studies did not record details about concealment and then we judged studies which reported concealment by a central office or an interactive third-party telephone via an interactive voice response system or web-based randomizations via interactive web-based response system as having no bias.  **Traditional meta-analysis**  We performed a series of conventional meta-analyses to summarize the classes of treatment regimens. OSR was better with T-DM1C than LC, with PEHC than HC (**Table 1**). ORR was better with PEHC than PEC and HC, with T-DM1C than LC. Direct comparisons of the safety of the 7 treatment regimens are in **Supplementary T1 Table 2**. We found no significant heterogeneity, with I^2^ <50% (**Table 1**). However, in side-effect analysis, we found significant heterogeneity in the meta-analysis of rash for the comparisons LC versus NST, LVEF (10%-50%) for HC versus NST, fatigue for HC versus NST, and diarrhea for LC versus HC (**Supplementary T1 Table 2**). For the meta-analysis of diarrhea, we found I^2^ values > 75% for the comparisons LC versus HC.  **Network meta-analysis**  The network of the 7 treatment regimens for ORR is in **Figure 2** and for OSR is in **Supplementary F2 Figure 3**. Of the 20 pair-wise comparisons between the 7 treatment regimens, 15 trials directly studied efficacy for OSR and ORR and 20 for safety.  In head-to-head comparisons, For OSR, the T-DM1C regimen was more effective than LC (OR 0.60 [95% CI 0.39, 0.94]). For ORR, the T-DM1C regimen was more effective than LC (OR 0.59 [0.40, 0.85]) and PEC (OR 0.45 [0.18, 0.91]), and the PEHC regimen was more effective than LC (OR 2.06 [1.29, 3.16]), HC (OR 1.85 [1.23, 2.67]), or PEC (OR 3.04 [1.48, 5.60]).  The results for safety for head-to-head comparisons are in **Supplementary T1 Table 3.** The LHC regimen was more associated with rash than the T-DM1C, HC, PEHC and NST regimens, and the PEHC regimen was less associated with rash than LC regimen. The LC regimen was less associated with diarrhea than the NC and HC regimens. We found no significant effects for LVEF, fatigue, nausea and vomiting, perhaps because of small sample size.  Most loops were consistent (see forest plots in **Supplementary F2 Figure 4**); one for rash for LC versus HC versus NST showed low inconsistency **(Supplementary F2 Figure 4c**), which implied that the direct estimate of the summary effect differed from the indirect estimate according to the forest plots.  **Supplementary T1 Table 5** presents 5 regimens ordered by their probability of being the best in terms of OSR as well as all HER2-targeted treatment regimens ordered by their probability of being the best in terms of ORR, showing the separate contributions to the overall scores of efficacy.  We performed a sensitive analysis and found that random- and fixed-effects models produced nearly the same results (**Supplementary T1 Table 6**). As well, many meta-analyses have focused on advanced or metastasized breast cancer, so we performed a subgroup analysis of HER2+ advanced breast cancer with the T-DM1C, LC, HC, NC, PEHC regimens (**Supplementary T1 Table 8**). For OSR, the T-DM1C regimen was more effective than LC, HC and NST. For ORR, the T-DM1C regimen was more effective than LC and NST, and the PEHC regimen was more effective than LC, HC and NST. Rash was more associated with LC than T-DM1C and more associated with LC than HC, and NST (**Supplementary T1 Table 9**). | #7- #9 |

Page 1 of 2

| **Section/topic** | **#** | **Checklist item** | **Reported on page #** |
| --- | --- | --- | --- |
| Risk of bias across studies | 15 | The risk of bias of trials was assessed as recommended by the Cochrane Collaboration, which was conducted on all 20 studies included for systematic review (**Supplementary F2 Figures 1-2**). | #5 |
| Additional analyses | 16 | We performed a subgroup analysis using a random-effects model of early and advanced HER2+ breast cancer. | #6 |
| **RESULTS** | | |  |
| Study selection | 17 | **Figure 1: Flow of articles in the study**  20 randomized trials corresponding to 43 groups because 3 were three-group studies. | #7 |
| Study characteristics | 18 | **Supplementary T1 Table 1: Characteristics of 20 included trials** | #7 |
| Risk of bias within studies | 19 | **Supplementary F2 Figure 4:** Most loops were consistent one for rash for LC versus HC versus NST showed low inconsistency **(Supplementary F2 Figure 4c**), which implied that the direct estimate of the summary effect differed from the indirect estimate according to the forest plots.  **Supplementary T1 Table 6**: We performed a sensitive analysis and found that random- and fixed-effects models produced nearly the same results. | #9 |
| Results of individual studies | 20 | **Figure 2**: The network of the 7 treatment regimens for ORR. For OSR is in **Supplementary F2 Figure 3**. Of the 20 pair-wise comparisons between the 7 treatment regimens, 15 trials directly studied efficacy for OSR and ORR and 20 for safety. | #8 |
| Synthesis of results | 21 | **Table 1:** OSR was better with HC and LC regimens than NST, with T-DM1C than LC, and PEHC than HC.  **Table 2:** In head-to-head comparisons, naïve standard treatment (NST) had the worst efficacy for OSR and ORR as compared with other HER2-targeted treatments. For OSR, the T-DM1C regimen was more effective than LC (OR 0.61 [95% CI 0.39, 0.94]) and NST (OR 0.44 [0.24, 0.73]). For ORR, the T-DM1C regimen was more effective than LC (OR 0.59 [0.40,0.85]), NST (OR 0.27 [0.17,0.43]), or PEC (OR 0.45 [0.18,0.91]), and the PEHC regimen was more effective than LC (OR 2.06 (1.29,3.16)), HC (OR 1.85 [1.23,2.67]), NST (OR 4.53 [2.79,6.94]), or PEC (OR 3.04 [1.48,5.60]).  **Supplementary T1 Table 3: T**he LHC regimen was more associated with rash than the T-DM1C, HC, PEHC and NST regimens, and the PEHC regimen was less associated with rash than the LC regimen. The LC regimen was less associated with diarrhea than the NC and HC regimens. We found no significant effects for LVEF, fatigue, nausea and vomiting, perhaps because of small sample size.  **Supplementary T1 Table 8-9:** we performed a subgroup analysis of HER2+ advanced breast cancer with the T-DM1C, LC, HC, NC, PEHC regimens. For OSR, the T-DM1C regimen was more effective than LC, HC and NST. For ORR, the T-DM1C regimen was more effective than LC and NST, and the PEHC regimen was more effective than LC, HC and NST. Rash was more associated with LC than T-DM1C and more associated with LC than HC, and NST. | #8 - #9 |
| Risk of bias across studies | 22 | **Supplementary F2 Figures 1-2:** The overall quality of studies was rated moderate. Some studies did not record details about concealment and we judged studies reporting concealment by a central office as having no bias. | #7 |
| Additional analysis | 23 | **Supplementary T1 Table 8:** we performed a subgroup analysis of HER2+ advanced breast cancer with the T-DM1C, LC, HC, NC, PEHC regimens. For OSR, the T-DM1C regimen was more effective than LC, HC and NST. For ORR, the T-DM1C regimen was more effective than LC and NST, and the PEHC regimen was more effective than LC, HC and NST.  **Supplementary T1 Table 9:** Rash was more associated with LC than T-DM1C and more associated with LC than HC, and NST. | #9 |
| **DISCUSSION** | | |  |
| Summary of evidence | 24 | In our network meta-analysis, the regimen of T-DM1 in combination with capecitabine as well as pertuzumab in combination with trastuzumab and docetaxel to some extent outperformed other treatment regimens. And the treatment effects were indistinguishable in magnitude. We found no difference in outcomes among the LC, HC, and LHC regimens for treatment of HER2+ breast cancer. In terms of side effects, rash was more associated with the LHC regimen than PEHC, HC, T-DM1C regimens, less associated with the PEHC than LC regimen. The PEC regimen did differ from other regimens perhaps because of limited sample size. Adding pertuzumab to standard treatment was previously found associated with a significant risk of rash. Furthermore, risk of abnormal LVEF was greater with adding trastuzumab to standard treatment than naïve standard treatment. Overall, no two HER2-targeted treatment regimens were significantly different about the remaining side effects.  With 7 treatment regimens for HER2+ breast cancer, direct comparisons are clearly limited by the relative small number of studies to evaluate a specific pair of treatment regimens. Network meta-analysis reduces this problem by using indirect comparisons and data synthesis to distinguish the most effective and safest treatment. Nonetheless, we found no meaningful data for the side effects of fatigue, diarrhea, vomiting and nausea.  A mixed model is thought to be the most appropriate for multiple-treatment meta-analysis . Except for a heterogeneity >75% for the LC versus HC regimens for the side effect diarrhea, we found little heterogeneity among all peer-comparisons. As well, the categories of HER2-targeted treatment might be the first attempt to be used to compare direct and indirect data and the network approaches for HER2-targeted treatment. Some network or conventional analyses have been published for advanced breast cancer, but they either just focused on targeted therapy without stressing the HER2-targeted therapy or did not use multiple-treatments meta-analysis.  Our evidence supports adding HER2-targeted agents to standard treatments for HER2+ advanced or metastatic breast cancer, but the optimal duration of ErbB-family receptor inhibitors added to standard treatment is not clear. In our multiple-treatments meta-analysis and pair-wise meta-analysis, OSR and ORR was associated more with the T-DM1C than LC regimen, HC and LC regimens than naive standard treatment (NST), and LHC regimen than LC and HC regimens but linked to rash and gastrointestinal disorders. In the subgroup analysis, because of the limited of sample sizes, we included T-DM1C, LC, HC, and PEHC as well as NST for analysis of OSR and all 7 treatment regimens for analysis of ORR. The best treatment for OSR was T-DM1C and PEHC for ORR. In our network analysis, we found T-DM1C and PEHC regimens as ideal HER2-targeted treatment regimens for treating HER2+ breast cancer and accompanied by attributable to moderate toxicity. | #10 |
| Limitations | 25 | Our study has some disadvantages. First, the network could not be expanded to unpublished trials and extracted published data rather than individual patient information. Secondly, we chose the suitable trials without consideration whether the trial is first-line or not because of lack of enough trials. Although the accuracy of the results might be misled in some extent, we had attempted to go through a subgroup analysis for first/second-line HER2+ advanced or metastatic breast cancer and got more reliable results. In addition, some trials were open-label and the concealment was not detailed clearly; thus, the validity of the results might be underestimated. Third, in any meta-analysis, selection bias and publication bias cannot be avoided. In subgroup analysis, we did not estimate the effect on early HER2+ breast cancer because only 4 trials were available on this topic, which is insufficient for constructing a closed loop. Finally, our study did not stress high- and low-dosing HER2-targeted agents combined with standard treatments. If we detail such treatment regimens like that, these regimens can not be connected with each other in a closed loop. In future, with the increasing number of clinical trials for HER2+ breast cancer, we wishes that we can provide details of HER2-targeted agents added to concrete standard treatments to provide meaningful data to clinicians and researchers in this field. | #11 |
| Conclusions | 26 | In conclusion, our study supports that the regimen of T-DM1 in combination with capecitabine as well as pertuzumab in combination with trastuzumab and docetaxel might be the best for HER2+ breast cancer in terms of overall survival rate (OSR) and overall response rate (ORR) and with moderate toxicity, especially for HER2+ advanced or metstatic breast cancer. Naive standard treatment (NST) without the HER2-targeted agents showed poor efficacy, but which with the least toxicity. The suggested hierarchies for the 7 treatment regimens should give some information for clinicians to choose a suitable targeted therapy for HER2+ breast cancer. We wish that more and more randomized trials, which consist of more than one HER2-targeted agents in combination with chemotherapy/hormone therapy in HER2-positive breast cancer, can be increased to represent various and efficacious treatment regimens. | #12 |
| **FUNDING** | | |  |
| Funding | 27 | No any funding and support. |  |

*From:*  Moher D, Liberati A, Tetzlaff J, Altman DG, The PRISMA Group (2009). Preferred Reporting Items for Systematic Reviews and Meta-Analyses: The PRISMA Statement. PLoS Med 6(6): e1000097. doi:10.1371/journal.pmed1000097

For more information, visit: **www.prisma-statement.org**.

Page 2 of 2
