# Supplementary material for: Efficacy and Safety of HER2-Targeted Agents for Breast Cancer with HER2-Overexpression: A Network Meta-Analysis
Source: PLoS One. 2015 May 20;10(5):e0127404. doi: 10.1371/journal.pone.0127404 (PMC4439018; doi:10.1371/journal.pone.0127404)
Supplement: S1 Table — (DOC) [file pone.0127404.s007.doc]

**S1 Table. S**earch strategy in PubMed

| Search | Query | Items found |
| --- | --- | --- |
| #1 | "breast neoplasms"[mesh] | 217296 |
| #2 | (breast or mammary) and (cancer* or tumour* or tumor* or neoplas* or carcinoma) | 308018 |
| #3 | # 1 and #2 | 308018 |
| #4 | trastuzumab or Herceptin or lapatinib or Tykerb or pertuzumab or Omnitarg | 7241 |
| #5 | #3 and #4 limits: clinical trials, human | 575 |

Search time limits: March 1st 2014
