# Supplementary material for: Efficacy and Safety of HER2-Targeted Agents for Breast Cancer with HER2-Overexpression: A Network Meta-Analysis
Source: PLoS One. 2015 May 20;10(5):e0127404. doi: 10.1371/journal.pone.0127404 (PMC4439018; doi:10.1371/journal.pone.0127404)
Supplement: S2 Table — (DOC) [file pone.0127404.s008.doc]

**S2 Table. Characteristics of included trials**

| **Study and year** | **Mean age (y)** | **No. of patients** | **HER2-targeted therapy** | **Standard treatment** | **Cycles of**  **therapeutic partner** | **Line of treatment** | **HER2 Status**  **Targeted identification at**  **Randomization** | **Diagnosis** | **Follow-up**  **(months)** |
| --- | --- | --- | --- | --- | --- | --- | --- | --- | --- |
| Slamon  2001 | 52.8 | 235 | Trastuzumab q1w | (anthracycline^c^**/** pacitaxel 175 mg/m^2^ q3w | 6 | first | IHC2+ or IHC3+ | HER2+MBC | 30 |
|  | 52.8 | 234 |  | (anthracycline^c^**/** pacitaxel 175 mg/m^2^ q3w | 6 |  |  |  |  |
| Marty  2005 | 53 | 92 | Trastuzumab q1w | Docetaxel 100 mg/m^2^ q3w | 6 | first | IHC3+ and/or FISH positive | HER2+MBC | 40.9 |
|  | 55 | 96 |  | Docetaxel 100 mg/m^2^ q3w | 6 |  |  |  |  |
| Gasparini  2007 | 56 | 63 | Trastuzumab q1w | Paclitaxel 80 mg/m^2^ q1w | Until progression | first | IHC2+ or IHC3+ | HER2+ABC | 16.6 |
|  | 54 | 60 |  | Paclitaxel 80 mg/m^2^ q1w | Until progression |  |  |  |  |
| Von Minckwitz 2009 | 53 | 78 | Trastuzumab q3w | Capecitabine 2500 mg/m^2^ day 1-14 q3w | Until progression | Second | IHC3+ and/or FISH positive | HER2+ABC or  MBC | 40 |
|  | 59 | 78 |  | Capecitabine 2500 mg/m^2^ day 1-14 q3w | Until progression |  |  |  |  |
| Kaufman  2009 | 56 | 103 | Trastuzumab q1w | Anastrozole 1 mg/m^2^ daily | Until progression | first | IHC3+ and /or FISH positive | HER2+MBC | 24 |
|  | 54 | 104 |  | Anastrozole 1 mg/m^2^ daily | Until progression |  |  |  |  |
| Huober  2011 | 60 | 26 | Trastuzumab q1w | Letrozole 2.5 mg/m^2^ daily | Until progression | first | IHC3+ or FISH positive | HER2+MBC | 18 |
|  | 61.5 | 31 |  | Letrozole 2.5 mg/m^2^ daily | Until progression |  |  |  |  |
| Verma  2012 | 53 | 495 | T-DM1 3.6 mg/m2  q21d |  | Until progression | second | IHC3+ and/or FISH positive | HER2+MBC | 36 |
|  | 53 | 496 | lapatinib | Capecitabine 1000 mg/m^2^ q2 d1-14 of 21-d | Until progression |  |  |  |  |
| Guan 2013 | 50 | 222 | Lapatinib 1500 mg/d | Paclitaxel 80 mg/m2 q4w | 6 | first | IHC2+ | HER2+MBC | 53 |
|  | 50.5 | 222 | Placebo | paclitaxel 80 mg/m2 q4w | 6 |  |  |  |  |
| Untch  2012 | 50 | 307 | Trastuzumab q3w | ^d^Epiruoicon q3w | 4 | first | IHC2+ or FISH positive | HER2+ABC | 24 |
|  | 50 | 308 | Lapatinib 1000 mg daily | ^d^Epiruoicon q3w | 4 |  |  |  |  |
| Cameron  2010 | 54 | 207 | Lapatinib 1250 mg daily | Capecitabine 2000 mg/m2 day1-14 q3w | Until progression | Second or sequence line | IHC3+ or IHC2+ or FISH positive | HER2+MBC | 34 |
|  | 51 | 201 |  | Capecitabine 2000 mg/m2 day1-14 q3w | Until progression |  |  |  |  |
| Schwartzberg 2010 | 60 | 111 | Lapatinib 1500 mg/d | Letrozole 2.5 mg/d | Until progression | first | IHC3+ or IHC2+ or FISH positive | HER2+MBC | 21.6 |
|  | 59 | 108 | placebo | Letrozole 2.5 mg/d | Until progression |  |  |  |  |
| Di Leo A  2008 | 51 | 49 | Lapatinib 1500 mg/d | Paclitaxel 175 mg/m2 q3w | 6 | first | IHC2+ or FISH  positive | HER2+MBC | 7 |
|  | 51 | 37 | placebo | Paclitaxel 175 mg/m2 q3w | 6 |  |  |  |  |
| Smith 2013 | 53.4 | 271 | Lapatinib 1250 mg/d | Capecitabine 2500 mg/m2 twice daily | Until progression | Second or sequence line | IHC2+ or FISH  positive | HER2+MBC | 10 |
|  | 55.8 | 269 | Trastuzumab q3w | Capecitabine 2500 mg/m2 twice daily | Until progression |  |  |  |  |
| Swain 2013 | 54 | 402 | Pertuzumab +trastuzumab q3w | Docetaxel 75 mg/m2 q3w | Until progression | first | IHC3+ or FISH  positive | HER2+MBC | 47 |
|  | 54 | 406 | Placebo+ trastuzuamb q3w | Docetaxel 75 mg/m2 q3w | Until progression |  |  |  |  |
| Gianni  2011 | 50 | 107 | Trastuzumab q3w | Docetaxel 75 mg/m² q3w | 4 | first | IHC3+ or IHC2+ or FISH positive | HER2+BC | 24 |
|  | 50 | 107 | Pertuzumab +trastuzumab q3w | Docetaxel 75 mg/m² q3w | 4 |  |  |  |  |
|  | 49 | 96 | Pertuzumab q3w | Docetaxel 75 mg/m² q3w | 4 |  |  |  |  |
| Azim  2013 | 50 | 154 | Lapatinib 1500 mg/d | Paclitaxel+ ^e^FEC | Not stated | unknown | been accredited by our certified  Laboratory (Vall d’Hebron Institute of Oncology). | HER2+EBC | 24 |
|  | 49 | 149 | Trastuzumab | Paclitaxel+^e^FEC | Not stated |  |  |  |  |
|  | 50 | 152 | Lapatinib 1,000 mg/day +trastuzumab | Paclitaxel+ ^e^FEC | Not stated |  |  |  |  |
| Robidoux  2013 | Not stated | 177 | Trastuzumab q3w | ^f^Paclitaxel q3w | 4 |  | IHC3+ or  FISH positive | HER2+EBC | 13 |
|  | Not stated | 159 | Lapatinib 1250 mg/d | ^f^Paclitaxel q3w | 4 |  |  |  |  |
|  | Not stated | 165 | Lapatinib 1000 mg/d +trastuzumab q3w | ^f^Paclitaxel q3w | 4 |  |  |  |  |
| Perez  2011 | Not stated | 1097 | Trasuzumab q3w | ^f^Paclitaxel q3w | Not stated | unknown | IHC3+ or  FISH positive | HER2+EBC | 72 |
|  | Not stated | 1087 |  | ^f^Paclitaxel q3w | Not stated |  |  |  |  |
| Slamon  2012 | Not stated | 1073 | Trastuzumab q3w | ^d^Epiruoicon q3w | 6 | unknown | FISH positive | HER2+EBC | 65 |
|  | Not stated | 1074 |  | ^d^Epiruoicon q3w | 6 |  |  |  |  |
| Joensuu  2006 | 51.4 | 115 | Trastuzumab | Vinorelbine 25 mg/m2 q21d + ^e^FEC | 3 | unknown | IHC3+ or IHC2+ or FISH positive | HER2+EBC | 37 |
|  | 49.9 | 116 |  | Vinorelbine 25 mg/m2 q21d + ^e^FEC | 3 |  |  |  |  |
| Hurvitz SA | 52 | 70 | traszutumab | Docetacel 75 or 100 mg/m2 | 12 | Second or sequence line | IHC3+ or  FISH positive | HER2+MBC | 14 |
|  | 52 | 67 | T-MD1 |  | 16 |  |  |  |  |

^c^Anthracycline, doxorubicin or epirubicin and cyclophosphoamide.

^d^Epiruoicon, epiruoicon and cylophosphamide and docetaxel

^e^FEC, fluorouracil and epirubicin and cyclophosphamide.

^f^Paclitaxel, doxorubicin and cyclophosphamide

trastuzuamb q3w: trastuzumab 8 mg/kg loading dose, 6 mg/kg maintenance; trastuzumab q1w: trastuzumab 4 mg/kg loading dose, 2 mg/kg maintenance

MBC: metastasis breast cancer; EBC: early breast cancer; ABC: advanced breast cancer; BC: breast cancer; T-DM1: trastuzumab emtansine; IHC: immunohistochemistry; FISH: fluorescence *in situ* hybridization.

1. Eiermann W, International Herceptin Study G (2001) Trastuzumab combined with chemotherapy for the treatment of HER2-positive metastatic breast cancer: pivotal trial data. Ann Oncol 12 Suppl 1: S57-62.

2. Slamon DJ, Leyland-Jones B, Shak S, Fuchs H, Paton V, et al. (2001) Use of chemotherapy plus a monoclonal antibody against HER2 for metastatic breast cancer that overexpresses HER2. N Engl J Med 344: 783-792.

3. Marty M, Cognetti F, Maraninchi D, Snyder R, Mauriac L, et al. (2005) Randomized phase II trial of the efficacy and safety of trastuzumab combined with docetaxel in patients with human epidermal growth factor receptor 2-positive metastatic breast cancer administered as first-line treatment: the M77001 study group. J Clin Oncol 23: 4265-4274.

4. Gasparini G, Gion M, Mariani L, Papaldo P, Crivellari D, et al. (2007) Randomized Phase II Trial of weekly paclitaxel alone versus trastuzumab plus weekly paclitaxel as first-line therapy of patients with Her-2 positive advanced breast cancer. Breast Cancer Res Treat 101: 355-365.

5. von Minckwitz G, du Bois A, Schmidt M, Maass N, Cufer T, et al. (2009) Trastuzumab beyond progression in human epidermal growth factor receptor 2-positive advanced breast cancer: a german breast group 26/breast international group 03-05 study. J Clin Oncol 27: 1999-2006.

6. Kaufman B, Mackey JR, Clemens MR, Bapsy PP, Vaid A, et al. (2009) Trastuzumab plus anastrozole versus anastrozole alone for the treatment of postmenopausal women with human epidermal growth factor receptor 2-positive, hormone receptor-positive metastatic breast cancer: results from the randomized phase III TAnDEM study. J Clin Oncol 27: 5529-5537.

7. Huober J, Fasching PA, Barsoum M, Petruzelka L, Wallwiener D, et al. (2012) Higher efficacy of letrozole in combination with trastuzumab compared to letrozole monotherapy as first-line treatment in patients with HER2-positive, hormone-receptor-positive metastatic breast cancer - results of the eLEcTRA trial. Breast 21: 27-33.

8. Verma S, Miles D, Gianni L, Krop IE, Welslau M, et al. (2012) Trastuzumab emtansine for HER2-positive advanced breast cancer. N Engl J Med 367: 1783-1791.

9. Guan Z, Xu B, DeSilvio ML, Shen Z, Arpornwirat W, et al. (2013) Randomized trial of lapatinib versus placebo added to paclitaxel in the treatment of human epidermal growth factor receptor 2-overexpressing metastatic breast cancer. J Clin Oncol 31: 1947-1953.

10. Untch M, Loibl S, Bischoff J, Eidtmann H, Kaufmann M, et al. (2012) Lapatinib versus trastuzumab in combination with neoadjuvant anthracycline-taxane-based chemotherapy (GeparQuinto, GBG 44): a randomised phase 3 trial. Lancet Oncol 13: 135-144.

11. Cameron D, Casey M, Oliva C, Newstat B, Imwalle B, et al. (2010) Lapatinib plus capecitabine in women with HER-2-positive advanced breast cancer: final survival analysis of a phase III randomized trial. Oncologist 15: 924-934.

12. Geyer CE, Forster J, Lindquist D, Chan S, Romieu CG, et al. (2006) Lapatinib plus capecitabine for HER2-positive advanced breast cancer. N Engl J Med 355: 2733-2743.

13. Johnston S, Pippen J, Jr., Pivot X, Lichinitser M, Sadeghi S, et al. (2009) Lapatinib combined with letrozole versus letrozole and placebo as first-line therapy for postmenopausal hormone receptor-positive metastatic breast cancer. J Clin Oncol 27: 5538-5546.

14. Schwartzberg LS, Franco SX, Florance A, O'Rourke L, Maltzman J, et al. (2010) Lapatinib plus letrozole as first-line therapy for HER-2+ hormone receptor-positive metastatic breast cancer. Oncologist 15: 122-129.

15. Di Leo A, Gomez HL, Aziz Z, Zvirbule Z, Bines J, et al. (2008) Phase III, double-blind, randomized study comparing lapatinib plus paclitaxel with placebo plus paclitaxel as first-line treatment for metastatic breast cancer. J Clin Oncol 26: 5544-5552.

16. Baselga J, Cortes J, Kim SB, Im SA, Hegg R, et al. (2012) Pertuzumab plus trastuzumab plus docetaxel for metastatic breast cancer. N Engl J Med 366: 109-119.

17. Swain SM, Ewer MS, Cortes J, Amadori D, Miles D, et al. (2013) Cardiac tolerability of pertuzumab plus trastuzumab plus docetaxel in patients with HER2-positive metastatic breast cancer in CLEOPATRA: a randomized, double-blind, placebo-controlled phase III study. Oncologist 18: 257-264.

18. Swain SM, Kim SB, Cortes J, Ro J, Semiglazov V, et al. (2013) Pertuzumab, trastuzumab, and docetaxel for HER2-positive metastatic breast cancer (CLEOPATRA study): overall survival results from a randomised, double-blind, placebo-controlled, phase 3 study. Lancet Oncol 14: 461-471.

19. Gianni L, Pienkowski T, Im YH, Roman L, Tseng LM, et al. (2012) Efficacy and safety of neoadjuvant pertuzumab and trastuzumab in women with locally advanced, inflammatory, or early HER2-positive breast cancer (NeoSphere): a randomised multicentre, open-label, phase 2 trial. Lancet Oncol 13: 25-32.

20. Azim HA, Jr., Agbor-Tarh D, Bradbury I, Dinh P, Baselga J, et al. (2013) Pattern of rash, diarrhea, and hepatic toxicities secondary to lapatinib and their association with age and response to neoadjuvant therapy: analysis from the NeoALTTO trial. J Clin Oncol 31: 4504-4511.

21. Robidoux A, Tang G, Rastogi P, Geyer CE, Jr., Azar CA, et al. (2013) Lapatinib as a component of neoadjuvant therapy for HER2-positive operable breast cancer (NSABP protocol B-41): an open-label, randomised phase 3 trial. Lancet Oncol 14: 1183-1192.

22. Perez EA, Suman VJ, Davidson NE, Gralow JR, Kaufman PA, et al. (2011) Sequential versus concurrent trastuzumab in adjuvant chemotherapy for breast cancer. J Clin Oncol 29: 4491-4497.

23. Slamon D, Eiermann W, Robert N, Pienkowski T, Martin M, et al. (2011) Adjuvant trastuzumab in HER2-positive breast cancer. N Engl J Med 365: 1273-1283.

24. Joensuu H, Kellokumpu-Lehtinen PL, Bono P, Alanko T, Kataja V, et al. (2006) Adjuvant docetaxel or vinorelbine with or without trastuzumab for breast cancer. N Engl J Med 354: 809-820.

25. Hurvitz SA, Dirix L, Kocsis J, Bianchi GV, Lu J, et al. (2013) Phase II randomized study of trastuzumab emtansine versus trastuzumab plus docetaxel in patients with human epidermal growth factor receptor 2-positive metastatic breast cancer. J Clin Oncol 31: 1157-1163.
