# Supplementary material for: Efficacy and Safety of HER2-Targeted Agents for Breast Cancer with HER2-Overexpression: A Network Meta-Analysis
Source: PLoS One. 2015 May 20;10(5):e0127404. doi: 10.1371/journal.pone.0127404 (PMC4439018; doi:10.1371/journal.pone.0127404)
Supplement: S3 Table — (DOC) [file pone.0127404.s009.doc]

**S3 Table. Conventional meta-analysis of direct comparisons for outcomes**

| **Comparisons** | **Overall survival rate** | | | | **Overall response rate** | | | |
| --- | --- | --- | --- | --- | --- | --- | --- | --- |
|  | **OR** | **95% CI** | **I ^2^** | **P** | **OR** | **95% CI** | **I ^2^** | **P** |
| HC vs NST | 1.39 | 1.11,1.73 | 39.6% | 0.128 | 2.80 | 2.02,3.90 | 28.0% | 0.225 |
| LC vs NST | 1.41 | 1.09,1.81 | 0% | 0.842 | 2.23 | 1.70,2.32 | 0% | 0.891 |
| LC vs HC | 0.92 | 0.53,1.59 | - | - | 0.88 | 0.68,1.14 | 0% | 0.392 |
| T-DM1 vs LC | 1.70 | 1.32,2.19 | - | - | 1.73 | 1.29,2.32 | - | - |
| T-DM1 VS HC | 0.99 | 0.84，1.16 | - | - | 1.05 | 0.52,2.09 | - | - |
| HC vs PEHC | 0.71 | 0.54,0.94 | - | - | 0.56 | 0.40,0.77 | 0% | 0.910 |
| HC vs LHC | - | - | - | - | 0.59 | 0.35,1.00 | - | - |
| PEC vs PEHC | - | - | - | - | 0.34 | 0.16,0.75 | - | - |
| HC vs PEC | - | - | - | - | 1.58 | 0.81,3.09 | - | - |
| LC vs LHC | - | - | - | - | 0.70 | 0.41,1.20 | - | - |

LC, lapatinib; HC, trastuzumab; NST, naïve standard treatment (without any-targeted agents);

PEHC, pertuzumab and trastuzumab;

PEC, pertuzumab; LHC, lapatinib and trastuzumab.

| **Comparisons** | **Rash** | | | | **LVEF** | | | |
| --- | --- | --- | --- | --- | --- | --- | --- | --- |
|  | **OR** | **95% CI** | **I ^2^** | **P** | **OR** | **95% CI** | **I ^2^** | **P** |
| HC vs NST | 1.41 | 0.78,2.58 | 38.3% | 0.198 | 2.62 | 1.77,3.87 | 58.8% | **0.033** |
| LC vs NST | 2.99 | 1.75,5.09 | 65.5% | 0.055 | 2.15 | 0.54,8.60 | 26.5% | 0.257 |
| LC vs HC | 3.29 | 2.55,4.24 | 9.6% | 0.345 | 0.23 | 0.04,1.36 | 0% | 0% |
| T-DM1 vs LC | 0.33 | 0.23,0.47 | - | - | - | - | - | - |
| HC vs PEHC | 0.63 | 0.48,0.83 | 0% | 0.478 | 1.11 | 0.24,5.16 | 50.9% | 0.154 |
| HC vs LHC | 0.22 | 0.15,0.35 | 0% | 0.682 | - | - | - | - |
| PEC vs PEHC | 1.10 | 0.59,2.05 | - | - | 0.37 | 0.04,3.57 | - | - |
| HC vs PEC | 0.68 | 0.04,1.29 | - | - | 0.88 | 0.05,14.22 | - | - |
| LC vs LHC | 0.96 | 0.69,1.34 | 0% | 0.629 | - | - | - | - |
| T-DM1 vs HC | - | - | - | - | 1.05 | 0.22,5.00 | - | - |

| **Comparisons** | **Fatigue** | | | | **Diarrhea** | | | |
| --- | --- | --- | --- | --- | --- | --- | --- | --- |
|  | **OR** | **95% CI** | **I ^2^** | **P** | **OR** | **95% CI** | **I ^2^** | **P** |
| HC vs NST | 1.61 | 0.77,3.37 | 64.2% | 0.039 | 1.66 | 1.14,2.40 | 0% | 0.492 |
| LC vs NST | 1.53 | 1.111,2.09 | 0% | 0.902 | 5.80 | 1.76,19.68 | 15.1% | 0.278 |
| LC vs HC | 1.09 | 0.81,1.47 | 35.5% | 0.199 | 4.45 | 0.96,20.68 | 88.8% | 0.000 |
| T-DM1 vs LC | 1.40 | 1.07,1.83 | - | - | 0.11 | 0.03,0.49 | - | - |
| HC vs PEHC | 1.40 | 1.00,1.96 | 21.4% | 0.259 | 0.52 | 0.23,1.18 | 0% | 0.650 |
| HC vs LHC | 0.80 | 0.57,1.13 | 0% | 0.480 | 0.06 | 0.02,0.16 | 0% | 0.974 |
| PEC vs PEHC | 0.94 | 0.50,1.77 | - | - | 0.73 | 0.20,2.68 | - | - |
| HC vs PEC | 1.12 | 0.60,2.09 | - | - | 0.89 | 0.22,3.67 | - | - |
| LC vs LHC | 1.09 | 0.71.1.67 | 35.6% | 0.213 | 0.80 | 0.51,1.25 | 0% | 0.450 |

| **Comparisons** | **Nausea** | | | | **Vomiting** | | | |
| --- | --- | --- | --- | --- | --- | --- | --- | --- |
|  | **OR** | **95% CI** | **I ^2^** | **P** | **OR** | **95%CI** | **I ^2^** | **P** |
| HC vs NST | 0.94 | 0.72,1.22 | 0% | 0.651 | 1.11 | 0.792,1.542 | 0% | 0.423 |
| LC vs NST | 0.20 | 0.02,1.79 | 0% | 0.699 | 0.94 | 0.240,3.701 | 0% | 0.719 |
| LC vs HC | 0.84 | 0.42,1.70 | 0% | 0.672 | 1.36 | 0.645,2.850 | 0% | 0.506 |
| T-DM1 vs LC | 0.33 | 0.03,3.21 | - | - | 0.66 | 0.235,1.850 | - | - |
| HC vs PEHC | 2.98 | 0.21,73.32 | - | - | 0.50 | 0.045,5.536 | - | - |
| HC vs LHC | 0.32 | 0.08,1.37 | 0% | 0.735 | 0.38 | 0.124,1.187 | 0% | 0.476 |
| PEC vs PEHC | - | - | - | - | - | - | - | - |
| HC vs PEC | - | - | - | - | - | - | - | - |
| LC vs LHC | 0.69 | 0.22,2.22 | 0% | 0.791 | 0.62 | 0.11,3.60 | 39.2% | 0.199 |

OR, odds ratio; 95% CI, 95% confidence interval; I^2^, I^2^ statistic; LC, lapatinib; HC, trastuzumab; NST, naïve standard treatment; PEC, pertuzumab; PEHC, pertuzumab and trastuzumab; LHC, lapatinib and trastuzumab; LVEF, left ventricular ejection fraction
