# Supplementary material for: Efficacy and Safety of HER2-Targeted Agents for Breast Cancer with HER2-Overexpression: A Network Meta-Analysis
Source: PLoS One. 2015 May 20;10(5):e0127404. doi: 10.1371/journal.pone.0127404 (PMC4439018; doi:10.1371/journal.pone.0127404)
Supplement: S4 Table — (DOC) [file pone.0127404.s010.doc]

**S4 Table. Safety of the 7 HER2-targeted treatment regimens in network meta-analysis**

| T-DM1 | **3.25**  **(1.52,6.14)** | 1.11  (0.48,2.23) | 0.85  (0.35,1.77) | 1.85  (0.57,4.58) | 1.73  (0.60,3.84) | **4.05**  **(1.58,8.86)** |  |
| --- | --- | --- | --- | --- | --- | --- | --- |
| 0.78  (0.30,1.76) | LC | **0.34**  **(0.24,0.47)** | 0.26  (0.17,0.36) | 0.57  (0.22,1.23) | **0.53**  **(0.28,0.92)** | 1.24  (0.73,2.10) |  |
| 0.84  (0.30,2.08) | 1.06  (0.69,1.62) | HC | 0.77  (0.50,1.12) | 1.68  (0.703,3.28) | 1.58  (0.89,2.44) | **3.71**  **(2.19,6.05)** |  |
| 0.59  (0.20,1.46) | 0.78  (0.44,1.16) | 0.73  (0.42,1.09) | NST | 2.27  (0.83,4.99) | **2.12**  **(1.01,4.02)** | **4.95**  **(2.54,8.30)** | **Rash** |
| 0.89  (0.16,2.64) | 1.11  (0.34,2.67) | 1.04  (0.35,2.44) | 1.51  (0.47,3.97) | PEC | 1.06  (0.45,2.09) | 2.58  (0.88,6.11) |  |
| 0.92  (0.25,2.70) | 1.20  (0.46,2.61) | 1.12  (0.52,2.24) | 1.65  (0.68,3.56) | 1.29  (0.43,3.11) | PEHC | **2.51**  **(1.21,4.88)** |  |
| 0.96  (0.24,2.78) | 1.18  (0.60,2.28) | 1.14  (0.57,2.06) | 1.64  (0.78,3.45) | 1.38  (0.37,3.65) | 1.17  (0.38,2.98) | LHC |  |
|  |  | **Fatigue** |  |  |  |  |  |

| LC | 2.42  (0.52,7.82) | 0.81  (0.19,2.41) | 2.59  (0.02,15.06) | 2.67  (0.27,11.97) |
| --- | --- | --- | --- | --- |
|  | HC | **0.36**  **(0.18,0.59)** | 0.99  (0.01,4.31) | 1.02  (0.26,3.34) |
|  |  | NST | 1.02  (0.26,3.34) | 3.43  (0.03,13.88) |
|  | **LVEF** |  | PEC | 3.32  (0.69,11.39) |
|  |  |  |  | PEHC |

| T-DM1 | 29.69  (0.90,153.65) | 7.76  (0.15,38.07) | 4.65  (0.08,26.81) | 25.16  (0.11,118.86) | 18.56  (0.18,111.03) | **77.07**  **(1.24,408.05)** |
| --- | --- | --- | --- | --- | --- | --- |
|  | LC | **0.26**  **(0.08,0.58)** | **0.16**  **(0.04,0.39)** | 0.81  (0.02,4.63) | 0.72  (0.06,2.90) | 2.29  (0.47,7.75) |
|  |  | HC | 0.67  (0.21,2.56) | 2.96  (0.14,15.29) | 2.89  (0.41,10.32) | **10.51**  **(1.84,38.72)** |
|  |  |  | NST | 6.07  (0.19,32.17) | 5.60  (0.56,23.98) | **19.43**  **(2.50,75.93)** |
|  | **Diarrhea** |  |  | PEC | 3.39  (0.15,15.81) | 21.86  (0.34,95.97) |
|  |  |  |  |  | PEHC | 7.16  (0.53,30.74) |
|  |  |  |  |  |  | LHC |

| T-DM1 | 4.37  (0.25,12.33) | 4.28  (0.13,18.78) | 4.64  (0.09,13.17) | 156.27  (0.07,321.10) | 82.67  (0.43,131.57) |  |
| --- | --- | --- | --- | --- | --- | --- |
| 21.66  (0.40,108.27) | LC | 0.95  (0.32,2.26) | 0.82  (0.19,2.28) | 25.59  (0.085,79.67) | 5.54  (0.58,20.31) |  |
| 31.93  (0.52,178.89) | 1.58  (0.60,3.66) | HC | 0.94  (0.27,2.08) | 29.09  (0.11,77.19) | 8.39  (0.77,26.29) | **Vomiting** |
| 39.47  (0.55,209.29) | 1.84  (0.59,4.78) | 1.20  (0.61,2.35) | NST | 47.35  (0.12,110.99) | 9.02  (0.76,52.93) |  |
| 60.07  (0.004,.355.75) | 7.26  (0.002,49.70) | 4.21  (0.002,32.79) | 3.80  (0.001,33.59) | PEHC | 20.04  (0.03,53.07) |  |
| **99.25**  **(1.02,567.70)** | 4.17  (0.89,11.93) | 1.20  (0.61,2.35) | 2.72  (0.50,8.04) | 2173.93  (0.07,1890.12) | LHC |  |
|  |  | **Nausea** |  |  |  |  |

Data are the odds ratios (ORs) and 95% credibility intervals (95% CI) in the column-defining treatment compared with those in the row-defining treatment. OR < 1 favors the column-defining treatment. To obtain ORs for comparisons in the opposite direction, reciprocals should be used (e g, the OR for T-DM1C compared with LC is 1/0.91=1.1). Significant results are in bold. OSR, overall survival rate; ORR, overall response rate
