# Supplementary material for: Efficacy and Safety of HER2-Targeted Agents for Breast Cancer with HER2-Overexpression: A Network Meta-Analysis
Source: PLoS One. 2015 May 20;10(5):e0127404. doi: 10.1371/journal.pone.0127404 (PMC4439018; doi:10.1371/journal.pone.0127404)
Supplement: S5 Table — (DOC) [file pone.0127404.s011.doc]

**S5 Table. Model fit in random-effects model and fixed-effects model**.

|  |  | toresdev | pD | DIC |
| --- | --- | --- | --- | --- |
| OSR | RE | 24.9415 | 20.1 | 188.3 |
|  | FE | 27.424 | 17.3 | 187.9 |
| ORR | RE | 26.6776 | 23.4 | 219.8 |
|  | FE | 26.629 | 22.2 | 218.6 |
| Rash incidence rate | RE | 30.4604 | 23.8 | 203.2 |
|  | FE | 35.372 | 19.3 | 203.6 |
| LVEF incidence rate | RE | 27.7662 | 21.2 | 145.5 |
|  | FE | 38.4733 | 16.3 | 151.3 |
| Fatigue incidence rate | RE | 38.0028 | 27.2 | 220.5 |
|  | FE | 49.880 | 20.0 | 225.2 |
| Diarrhea incidence rate | RE | 29.4788 | 26.8 | 170.4 |
|  | FE | 56.0353 | 19.7 | 189.8 |
| Nausea incidence rate | RE | 23.6492 | 17.3 | 114.5 |
|  | FE | 23.1984 | 16.1 | 112.8 |
| Vomiting incidence rate | RE | 30.0807 | 20.6 | 127.3 |
|  | FE | 32.473 | 17.5 | 126.6 |

For each primary and second outcome we present toresdev, the number of data points and the deviance information criterion (DIC). The mean posterior deviance should approximate the number of data points for models with a good fit to the data. The DIC measures model fit penalized for complexity; smaller DIC values correspond to more parsimonious (and thus preferable) models. RE, random-effect model; FE, fixed-effect model; OSR, overall survival rate; ORR, overall response rate
