# Supplementary material for: Efficacy and Safety of HER2-Targeted Agents for Breast Cancer with HER2-Overexpression: A Network Meta-Analysis
Source: PLoS One. 2015 May 20;10(5):e0127404. doi: 10.1371/journal.pone.0127404 (PMC4439018; doi:10.1371/journal.pone.0127404)
Supplement: S6 Table — (DOC) [file pone.0127404.s012.doc]

**S6 Table. Ranking for efficacy and safety with random-effects** models

|  |  | **OSR** | **ORR** | **Rash** | **LVEF** | **Fatigue** | **Diarrhea** | **Vomiting** | **Nausea** |
| --- | --- | --- | --- | --- | --- | --- | --- | --- | --- |
| **Rank** | **T-DM1** | 1.31(1.00,3.00) | 2.21 (1.00,4.00) | 2.62(2.00,5.00) | NA | 5.73(1.00,7.00) | 2.01(1.00,6.00) | 2.48(1.00,6.00) | 2.70(1.00,5.00) |
|  | **LC** | 3.32(2.00-4.00) | 4.91(4.00,6.00) | 6.15(5.00,7.00) | 2.85(1.00,5.00) | 3.82(2.00,6.00) | 5.81(4.00,7.00) | 3.58(1.00,6.00) | 2.93(1.00,5.00) |
|  | **HC** | 3.47(2.00-4.00) | 4.18(3.00,5.00) | 2.60(1.00,4.00) | 4.27(3.00,5.00) | 4.09(2.00,7.00) | 3.20(2.00,5.00) | 2.95(1.00,5.00) | 3.84(2.00,5.00) |
|  | NST | 4.98(5.00-5.00) | 6.88(6.00,7.00) | 1.36(1.00,3.00) | 1.81(1.00,3.00) | 1.80(1.00,5.00) | 2.09(1.00,4.00) | 2.41(1.00,5.00) | 4.33(2.00,6.00) |
|  | **PEC** | NA | 5.87(4.00,7.00) | 4.25(1.00,6.00) | 2.49(1.00,5.00) | 3.65(1.00,7.00) | 3.70(1.00,7.00) | NA | NA |
|  | **PEHC** | 1.92(1.00-4.00) | 1.56(1.00,3.00) | 4.30(2.00,5.00) | 3.57(1.00,5.00) | 4.33(3.00,7.00) | 4.60(2.00,7.00) | 4.39(1.00,6.00) | 2.68(1.00,6.00) |
|  | **LHC** | NA | 2.43(1.00,5.00) | 6.73(5.00,7.00) | NA | 4.57(1.00,7.00) | 6.59(4.00,7.00) | 5.18(2.00,6.00) | 5.52(3.00,6.00) |
| **Best** | **T-DM1** | 0.74(0.00,1.00) | 0.00(0.00,0.00) | 0.20(0.00,1.00) | NA | 0.05(0.00,1.00) | 0.57(0.00,1.00) | 0.42(0.00,1.00) | 0.57(0.00,1.00) |
|  | **LC** | 0.00(0.00,0.00) | 0.00(0.00,0.00) | 0.00(0.00,0.00) | 0.15(0.00,1.00) | 0.03(0.00,0.00) | 0.00(0.00,0.00) | 0.04(0.00,1.00) | 0.034(0.00,1.00) |
|  | **HC** | 0.004(0.00,0.00) | 0.00(0.00,0.00) | 0.03(0.00,1.00) | 0.00(0.00,0.00) | 0.01(0.00,0.00) | 0.02(0.00,0.00) | 0.09(0.00,1.00) | 0.012(0.00,0.00) |
|  | NST | 0.00(0.00,0.00) | 0.00(0.00,0.00) | 0.72(0.00,1.00) | 0.38(0.00,1.00) | 0.51(0.00,1.00) | 0.28(0.00,1.00) | 0.27(0.00,1.00) | 0.006(0.00,0.00) |
|  | **PEC** | NA | 0.00(0.00,0.00) | 0.03(0.00,1.00) | 0.44(0.00,1.00) | 0.27(0.00,1.00) | 0.12(0.00,1.00) | NA | NA |
|  | **PEHC** | 0.25(0.00,1.00) | 0.58(0.00,1.00) | 0.007(0.00,0.00) | 0.03(0.00,1.00) | 0.07(0.00,1.00) | 0.002(0.00,0.00) | 0.17(0.00,1.00) | 0.37(0.00,1.00) |
|  | **LHC** | NA | 0.20(0.00,1.00) | 0.00\(0.00,0.00) | NA | 0.06(0.00,1.00) | 0.00(0.00,0.00) | 0.01(0.00,0.00) | 0.003(0.00,0.00) |

Best, probability of being the best. NA, no available data or zero incidence events is in two groups.
