# Supplementary material for: Efficacy and Safety of HER2-Targeted Agents for Breast Cancer with HER2-Overexpression: A Network Meta-Analysis
Source: PLoS One. 2015 May 20;10(5):e0127404. doi: 10.1371/journal.pone.0127404 (PMC4439018; doi:10.1371/journal.pone.0127404)
Supplement: S7 Table — (DOC) [file pone.0127404.s013.doc]

**S7 Table. Sensitivity analysis with fixed-effect models on the efficacy and safety of 6 regimens in network meta-analysis**

| T-DM1 | 0.60  (0.47,0.76) | 0.58  (0.39.0.80) | 0.42  (0.52,1.24) | 0.83  (0.73,1.27) |
| --- | --- | --- | --- | --- |
|  | LC | 0.97  (0.73,1.27) | 0.71  (0.55,0.90) | 1.39  (0.93,2.03) |
| **OSR** |  | HC | 0.73  (0.62,0.85) | 1.43  (1.07,1.84) |
|  |  |  | NST | 1.97  (1.41,2.67) |
|  |  |  |  | PEHC |

| T-DM1 | 0.58  (0.44,0.77) | 0.65  (0.45,0.91) | 0.27  (0.19,0.38) | 0.43  (0.20,0.81) | 1.20  (0.72,1.83) | 0.99  (0.54,1.65) |
| --- | --- | --- | --- | --- | --- | --- |
|  | LC | 1.12  (0.90,1.37) | 0.46  (0.37,0.57) | 0.74  (0.37,1.34) | 2.05  (1.40,2.95) | 1.70  (1.01,2.80) |
|  |  | HC | 0.42  (0.34,0.51) | 0.66  (0.35,1.19) | 1.84  (1.32,2.55) | 1.53  (0.93,2.46) |
|  |  |  | NST | 1.61  (0.79,2.92) | 4.48  (3.03,6.55) | 3.72  (2.24,6.16) |
|  |  | **ORR** |  | PEC | 3.05  (1.45,5.51) | 2.57  (1.05,5.28) |
|  |  |  |  |  | PEHC | 0.85  (0.46,1.46) |
|  |  |  |  |  |  | LHC |

| T-DM1 | 16.58  (2.97,73.29) | 5.01  (0.82,22.76) | 2.99  (0.47,12.55) | 7.74  (0.56,40.85) | 10.78  (1.34,45.16) | 27.40  (4.22,117.09) |  |
| --- | --- | --- | --- | --- | --- | --- | --- |
| 3.25  (1.52,6.32) | LC | 0.30  (0.20,0.44) | 0.18  (0.11,0.26) | 0.48  (0.08,1.49) | 0.67  (0.26,1.51) | 1.68  (1.07,2.51) |  |
| 1.11  (0.48,2.23) | 0.34  (0.24,0.47) | HC | 0.60  (0.42,0.93) | 1.59  (0.31,4.79) | 2.22  (0.93,4.88) | 5.66  (3.32,8.98) | **Diarrhea** |
| 0.85  (0.35,1.77) | 0.26  (0.17,0.36) | 0..77  (0.50,1.12) | NST | 2.71  (0.52,8.36) | 3.78  (1.43,8.93) | 9.65  (5.27,16.30) |  |
| 1.85  (0.57,4.58) | 0.57  (0.22,1.23) | 1.68  (0.70,3.28) | 2.27  (0.83,4.99) | PEC | 1.94  (0.51,5.60) | 5.65  (1.06,19.05) |  |
| 1.73  (0.60,6.84) | 0.53  (0.28,0.92) | 1.58  (0.89,2.44) | 2.12  (1.02,4.02) | 1.06  (0.45,2.09) | PEHC | 3.08  (0.99,6.91) |  |
| 4.05  (1.58,8.86) | 1.243  (0.73,2.10) | 3.71  (2.19,6.05) | 4.95  (2.54,8.30) | 2.58  (0.88,6.11) | 2.51  (1.21,4.88) | LHC |  |
|  |  | **Rash** |  |  |  |  |  |

| T-DM1 | 0.72  (0.54,0.93) | 0.71  (0.50,0.95) | 0.58  (0.39,0.82) | 0.69  (0.35,1.29) | 0.76  (0.49,1.11) | 0.77  (0.50,1.13) |
| --- | --- | --- | --- | --- | --- | --- |
|  | LC | 0.99  (0.80,1.19) | 0.80  (0.61,1.01) | 0.96  (0.51,1.74) | 1.06  (0.74,1.44) | 1.07  (0.79,1.41) |
|  |  | HC | 0.82  (0.62,1.08) | 0.98  (0.55,1.68) | 1.07  (0.82,1.38) | 1.09  (0.80,1.47) |
|  |  |  | NST | 1.22  (0.63,2.22) | 1.334  (0.89,1.92) | 1.36  (0.91,1.95) |
|  |  |  |  | PEC | 1.18  (0.65,1.95) | 1.22  (0.62,2.18) |
|  |  | **Fatigue** |  |  | PEHC | 1.04  (0.68,1.49) |
|  |  |  |  |  |  | LHC |

| LC | 1.81  (0.58,4.21) | 0.78  (0.26,1.84) | 1.21  (0.02,7.63) | 1.22  (0.31,3.32) |
| --- | --- | --- | --- | --- |
|  | HC | 0.43  (0.36,0.52) | 0.62  (0.01,3.25) | 0.67  (0.36,1.19) |
|  |  | NST | 1.45  (0.03,8.01) | 1.57  (0.81,2.86) |
|  | **LVEF** |  | PEC | 9.74  (0.20,56.66) |
|  |  |  |  | PEHC |

| T-DM1 | 1.78  (0.55,4.67) | 1.54  (0.36,4.76) | 1.40  (0.31,4.46) | 14.87  (0.19,94.36) | 3.40  (0.62,11.69) |  |
| --- | --- | --- | --- | --- | --- | --- |
| 11.53  (0.41,64.32) | LC | 0.85  (0.42,1.56) | 0.78  (0.37,1.43) | 8.62  (0.16,59.84) | 1.92  (0.69,4.17) | **Vomiting** |
| 16.08  (0.50,91.30) | 1.43  (0.73,2.62) | HC | 0.92  (0.66,1.27) | 10.29  (0.20,70.13) | 2.37  (0.83,5.34) |  |
| 17.36  (0.51,98.50) | 1.57  (0.75,3.00) | 1.10  (0.83,1.39) | NST | 11.36  (0.21,84.64) | 2.64  (0.87,6.17) |  |
| 67.12  (0.003,413.14) | 6.33  (0.001,31.47) | 4.18  (0.001,23.60) | 3.96  (0.001,22.44) | PEHC | 2.04  (0.03,11.96) |  |
| 40.66  (0.88,249.27) | 3.60  (0.91,9.43) | 2.63  (0.69,7.36) | 2.43  (0.62,6.91) | 60161  (0.0844,2948) | LHC |  |
|  |  | **Nausea** |  |  |  |  |

Data are the odds ratios (ORs) and 95% credibility intervals (95% CI) in the column-defining treatment compared with those in the row-defining treatment. OR < 1 favors the column-defining treatment. To obtain ORs for comparisons in the opposite direction, reciprocals should be used (e g, the OR for T-DM1C compared with LC is 1/0.91=1.1). Significant results are in bold.
