# Supplementary material for: Efficacy and Safety of HER2-Targeted Agents for Breast Cancer with HER2-Overexpression: A Network Meta-Analysis
Source: PLoS One. 2015 May 20;10(5):e0127404. doi: 10.1371/journal.pone.0127404 (PMC4439018; doi:10.1371/journal.pone.0127404)
Supplement: S8 Table — (DOC) [file pone.0127404.s014.doc]

**S8 Table. Ranking for efficacy and safety using fixed-effects models**

|  |  | **OSR** | **ORR** | **Rash** | **LVEF** | **Fatigue** | **Diarrhea** | **Vomiting** | **Nausea** |
| --- | --- | --- | --- | --- | --- | --- | --- | --- | --- |
| **Rank** | **T-DM1** | 1.19(1.00,2.00) | 2.19(1.00,3.00) | 2.43(1.00,4.00) | NA | 6.71(5.00,7.00) | 1.36(1.00,4.00) | 2.31(1.00,6.00) | 1.74(1.00,5.00) |
|  | **LC** | 3.34(2.00-4.00) | 4.93(4.00,6.00) | 6.14(6.00,7.00) | 3.16(1.00,5.00) | 3.92(2.00,6.00) | 5.81(4.00,6.00) | 3.77(2.00,5.00) | 2.87(1.00,5.00) |
|  | **HC** | 3.60(3.00-4.00) | 4.20(3.00,5.00) | 2.60(2.00,4.00) | 4.56(3.00,5.00) | 3.59(2.00,6.00) | 3.33(2.00,4.972) | 2.97(1.00,5.00) | 3.81(2.00,5.00) |
|  | NST | 5.00(5.00-5.00) | 6.90(6.00,7.00) | 1.17(1.00,2.00) | 1.91(1.00,3.00) | 1.59(1.00,4.00) | 1.92(1.00,3.00) | 2.30(1.00,5.00) | 4.37(2.00,6.00) |
|  | **PEC** | NA | 5.86(4.00,7.00) | 4.35(2.00,5.00) | 2.21(1.00,5.00) | 3.22(1.00,7.00) | 3.77(1.00,6.00) | NA | NA |
|  | **PEHC** | 1.87(1.00-3.00) | 1.50(1.00,3.00) | 4.48(4.00,5.00) | 3.15(1.00,5.00) | 4.41(3.00,7.00) | 4.87(4.00,6.00) | 4.53(1.00,6.00) | 2.66(1.00,6.00) |
|  | **LHC** | NA | 2.42(1.00,4.00) | 6.84(6.00,7.00) | NA | 4.57(1.00,7.00) | 6.94(6.00,7.00) | 5.13(2.00,6.00) | 5.55(3.00,6.00) |
| **Best** | **T-DM1** | 0.81(0.00,1.00) | 0.20(0.00,1.00) | 0.12(0.00,1.00) | NA | 0.00(0.00,0.00) | 0.77(0.00,1.00) | 0.48(0.00,1.00) | 0.53(0.00,1.00) |
|  | **LC** | 0.00(0.00,0.00) | 0.00(0.00,0.00) | 0.00(0.00,0.00) | 0.11(0.00,1.00) | 0.013(0.00,0.00) | 0.00(0.00,0.00) | 0.02(0.00,0.00) | 0.04(0.00,1.00) |
|  | **HC** | 0.00(0.00,0.00) | 0.00(0.00,0.00) | 0.014(0.00,0.00) | 0.00(0.00,0.00) | 0.014(0.00,0.00) | 0.00(0.00,0.00) | 0.07(0.00,1.00) | 0.0110(0.00,0.0) |
|  | NST | 0.00(0.00,0.00) | 0.00(0.00,0.00) | 0.85(0.00,1.00) | 0.29(0.00,1.00) | 0.59(0.00,1.00) | 0.19(0.00,1.00) | 0.26(0.00,1.00) | 0.002(0.00,0.00) |
|  | **PEC** | NA | 0.003(0.00,0.00) | 0.014(0.00,0.00) | 0.55(0.00,1.00) | 0.32(0.00,1.00) | 0.04(0.00,1.00) | NA | NA |
|  | **PEHC** | 0.19(0.00,1.00) | 0.61(0.00,1.00) | 0.00(0.00,0.00) | 0.046(0.00,1.00) | 0.03(0.00,1.00) | 0.001(0.00,0.00) | 0.15(0.00,1.00) | 0.41(0.00,1.00) |
|  | **LHC** | NA | 0.19(0.00,1.00) | 0.00(0.00,0.00) | NA | 0.04(0.00,1.00) | 0.00(0.00,0.00) | 0.02(0.00,0.00) | 0.004(0.00,0.00) |
