# Supplementary material for: Efficacy and Safety of HER2-Targeted Agents for Breast Cancer with HER2-Overexpression: A Network Meta-Analysis
Source: PLoS One. 2015 May 20;10(5):e0127404. doi: 10.1371/journal.pone.0127404 (PMC4439018; doi:10.1371/journal.pone.0127404)
Supplement: S9 Table — (DOC) [file pone.0127404.s015.doc]

**S9 Table. Subgroup analysis with random-effects models on efficacy and safety of HER2+ advance or metastatic breast cancer (OR 95%CI)**

| **T-DM1** | **0.61**  **(0.37,0.99)** | **0.60**  **(0.32,0.63)** | **0.44**  **(0.25,0.78)** | 0.87  (0.37,1.83) |  |
| --- | --- | --- | --- | --- | --- |
| **0.58**  **(0.38,0.87)** | **LC** | 0.99  (0.65,1.42) | **0.73**  **(0.53,0.99)** | 0.1.43  (0.74,2.47) |  |
| 0.68  (0.40,1.12) | 1.17  (0.19,1.60) | **HC** | 0.74  (0.57,1.00) | 1.44  (0.86,2.25) | **OSR** |
| **0.27**  **(0.16,0.43)** | **0.47**  **(0.36,0.61)** | **0.41**  **(0.31,53)** | NST | **1.98**  **(1.13,3.19)** |  |
| 1.25  (0.57,2.39) | **2.14**  **(1.15,3.70)** | **1.83**  **(1.04,2.87)** | **4.58**  **(2.50,7.48)** | **PEHC** |  |
|  |  | **ORR** |  |  |  |

| **T-DM1** | **2.85**  **(1.33,4.79)** | 1.12  (0.49,2.09) | 0.78  (0.32,1.49) | 2.39  (0.58,4.95) |
| --- | --- | --- | --- | --- |
|  | **LC** | **0.39**  **(0.23,0.62)** | **0.28**  **(0.17,0.45)** | 0.72  (0.25,1.72) |
| **Rash** |  | **HC** | 0.73  (0.20,1.18) | 1.84  (0.73,3.98) |
|  |  |  | NST | 2.75  (0.93,6.58) |
|  |  |  |  | **PEHC** |

| **LC** | 4.4134  (0.6817,13.2875) | 0.8193  (0.1756,2.2310) | 7.2261  (0.1346,13.1483) |
| --- | --- | --- | --- |
|  | **HC** | 0.2454  (0.0646,0.5759) | 1.3651  (0.0792,3.2349) |
| **LVEF** |  | NST | 8.6087  (0.2986,22.3791) |
|  |  |  | **PEHC** |

Data are the odds ratios (ORs) and 95% credibility intervals (95% CI) in the column-defining treatment compared with those in the row-defining treatment. OR < 1 favors the column-defining treatment. To obtain ORs for comparisons in the opposite direction, reciprocals should be used (e.g., the OR for T-DM1C compared with LC is 1/0.91=1.1). Significant results are in bold.
