# Supplementary material for: Efficacy and Safety of HER2-Targeted Agents for Breast Cancer with HER2-Overexpression: A Network Meta-Analysis
Source: PLoS One. 2015 May 20;10(5):e0127404. doi: 10.1371/journal.pone.0127404 (PMC4439018; doi:10.1371/journal.pone.0127404)
Supplement: S10 Table — (DOC) [file pone.0127404.s016.doc]

**S10 Table.** Ranking for efficacy and safety with fixed-effects models in subgroup analysis

|  |  | **OSR** | **ORR** | **Rash** | **LVEF** |
| --- | --- | --- | --- | --- | --- |
| **Rank** | **T-DM1** | 1.32(1.00,3.00) | 1.75(1.00,3.00) | 2.43(1.00,4.00) |  |
|  | **LC** | 3.33(2.00,4.00) | 3.79(3.00,4.00) | 4.84(4.00,5.00) | 2.07(1.00,4.00) |
|  | **HC** | 3.50(2.00,4.00) | 3.11(2.00,4.00) | 2.57(1.00,4.00) | 3.80(3.00,5.00) |
|  | NST | 4.93(4.00,5.00) | 5.00(5.00,5.00) | 1.27(1.00,3.00) | 1.36(1.00,3.00) |
|  | **PEC** | NA | NA | NA | NA |
|  | **PEHC** | 1.93(1.00,4.00) | 1.35(1.00,2.00) | 3.90(2.00,5.00) | 2.76(1.00,4.00) |
| **Best** | **T-DM1** | 0.74(0.00,1.00) | 0.31(0.00,1.00) | 0.15(0.00,1.00) | NA |
|  | **LC** | 0.004(0.00,0.00) | 0.001(0.00,0.00) | 0.00(0.00,0.00) | 0.22(0.00,1.00) |
|  | **HC** | 0.001(0.00,0.00) | 0.002(0.00,0.00) | 0.04(0.00,1.00) | 0.001(0.00,0.00) |
|  | NST | 0.001(0.00,0.00) | 0.00(0.00,0.00) | 0.79(0.00,1.00) | 0.68(0.00,1.00) |
|  | **PEC** | NA | NA | NA | NA |
|  | **PEHC** | 0.25(0.00,1.00) | 0.68(0.00,1.00) | 0.03(0.00,0.00) | 0.09(0.00,1.00) |
